# Characterisation of the antibody-mediated selective pressure driving intra-host evolution of SARS-CoV-2 in prolonged infection

**DOI:** 10.1371/journal.ppat.1012624

**Published:** 2024-10-15

**Authors:** Michael Schoefbaenker, Theresa Günther, Eva Ulla Lorentzen, Marie-Luise Romberg, Marc Tim Hennies, Rieke Neddermeyer, Marlin Maybrit Müller, Alexander Mellmann, Chiara Robin Bojarzyn, Georg Lenz, Matthias Stelljes, Eike Roman Hrincius, Richard Vollenberg, Stephan Ludwig, Phil-Robin Tepasse, Joachim Ewald Kühn

**Affiliations:** 1 Institute of Virology Muenster, University of Muenster, Muenster, Germany; 2 Institute of Hygiene, University Hospital Muenster, University of Muenster, Muenster, Germany; 3 Department of Medicine A, Haematology, Oncology and Pneumology, University Hospital Muenster, Muenster, Germany; 4 Department of Medicine B for Gastroenterology, Hepatology, Endocrinology and Clinical Infectiology, University Hospital Muenster, Muenster, Germany; Leiden University Medical Center: Leids Universitair Medisch Centrum, NETHERLANDS, KINGDOM OF THE

## Abstract

Neutralising antibodies against the SARS-CoV-2 spike (S) protein are major determinants of protective immunity, though insufficient antibody responses may cause the emergence of escape mutants. We studied the humoral immune response causing intra-host evolution in a B-cell depleted, haemato-oncologic patient experiencing clinically severe, prolonged SARS-CoV-2 infection with a virus of lineage B.1.177.81. Following bamlanivimab treatment at an early stage of infection, the patient developed a bamlanivimab-resistant mutation, S:S494P. After five weeks of apparent genetic stability, the emergence of additional substitutions and deletions within the N-terminal domain (NTD) and the receptor binding domain (RBD) of S was observed. Notably, the composition and frequency of escape mutations changed in a short period with an unprecedented dynamic. The triple mutant S:Delta141-4 E484K S494P became dominant until virus elimination. Routine serology revealed no evidence of an antibody response in the patient. A detailed analysis of the variant-specific immune response by pseudotyped virus neutralisation test, surrogate virus neutralisation test, and immunoglobulin-capture enzyme immunoassay showed that the onset of an IgM-dominated antibody response coincided with the appearance of escape mutations. The formation of neutralising antibodies against S:Delta141-4 E484K S494P correlated with virus elimination. One year later, the patient experienced clinically mild re-infection with Omicron BA.1.18, which was treated with sotrovimab and resulted in an increase in Omicron-reactive antibodies. In conclusion, the onset of an IgM-dominated endogenous immune response in an immunocompromised patient coincided with the appearance of additional mutations in the NTD and RBD of S in a bamlanivimab-resistant virus. Although virus elimination was ultimately achieved, this humoral immune response escaped detection by routine diagnosis and created a situation temporarily favouring the rapid emergence of various antibody escape mutants with known epidemiological relevance.

## Author summary

In the immunocompromised host, prolonged infection and insufficient antibody responses favour the emergence of highly mutated SARS-CoV-2 variants. To better understand the selective pressure exerted by antiviral antibodies, we characterised the variant-specific humoral immune response and resulting intra-host evolution of SARS-CoV-2 in a B-lymphocyte-depleted patient experiencing severe infection.

Treatment with a monoclonal antibody early in infection led to the selection and outgrowth of resistant virus. As their B-cell function recovered, the patient mounted an IgM-dominated antiviral antibody response, the onset of which closely correlated with the appearance of additional escape mutations within the spike protein. The strength and breadth of this antibody response gradually increased, resulting in the selection of a variant carrying several escape mutations within spike. The formation of neutralising antibodies against this variant resulted in virus elimination.

Our study provides new insights into the selection process of viral escape mutants in immunocompromised patients. The resuming humoral immunity against SARS-CoV-2 can be dominated by IgM. This immune response is poorly detectable with routine diagnostic procedures yet may trigger and drive substantial viral intra-host evolution. Determination of antiviral antibody responses and correlating them with intra-host evolution may provide a better picture of the adaptability of currently circulating SARS-CoV-2 variants.

## Introduction

More than three years into the severe acute respiratory syndrome coronavirus type 2 (SARS-CoV-2) pandemic, with a death toll of at least seven million people out of about 700 million diagnosed with coronavirus disease 2019 (COVID-19), the World Health Organization (WHO) declared the end of the health emergency state in May 2023. A rapid succession of viral immune escape variants has led to the dominance of Omicron and its descendants [1]. Currently circulating Omicron variants efficiently overcome immunity in vaccinees and convalescents and render numerous therapeutically relevant monoclonal antibodies ineffective [2]. Hybrid immunity resulting from previous vaccinations and infections appears to contribute to Omicron causing less severe disease than its predecessors [3,4]. Nevertheless, the high disease burden and lost working hours demand close monitoring of further viral evolution and gaining a deeper insight into the mechanisms of variant formation.

In general, the intra-host divergence of SARS-CoV-2 during acute infection was found to be low due to the combined effects of strong purifying selection and narrow transmission bottlenecks [5,6]. Among several hypotheses, such as unnoticed spread within distinct human populations and reciprocal transmission between an animal reservoir and humans, the persistence of infection in immunocompromised patients has been put front and centre in explaining the seemingly erratic emergence of heavily mutated escape variants, as reviewed by Markov et al. [7]. In the immunocompromised host, persistently high viral load, compartmentalisation, inadequate B and T cell immune responses, effects of the innate immunity, antibody-based therapeutic (ABT) measures exerting immunological pressure, and antiviral drugs eliciting mutations are supposed to alleviate the selection of escape variants [8–17]. The suppression of the B and T cell response, as observed in advanced HIV disease or severely immunosuppressed patients, is associated with the highest risk of prolonged infection [18,19]. Notably, prolonged infection may not be confined to immunosuppressed individuals. Estimates indicate a prevalence in the general population of approximately 1:1000 [20]. In a total of 2% of hospitalised patients experiencing virus persistence for two weeks or longer, the emergence of variants with multiple spike (S) mutations, predominantly affecting the spike subunit S1 region, was reported [21]. Strong intra-host evolution appears to be restricted to a specific subset of immunocompromised patients [22], with a greater prevalence detected in those severely immunocompromised [19].

While S-specific neutralising antibodies confer protection against severe disease and, albeit to a lesser extent, against re-infection, they simultaneously contribute to the evolution of escape variants [23–29]. The mutations associated with prolonged infections are frequently high-fitness antibody escape mutations within S1, which may indicate future evolutionary trends and the potential global success of SARS-CoV-2 variants [30]. In breakthrough infection, antibody responses were found to be dominated by immune imprinting due to previous vaccination or infection, which may induce convergent evolution of hot spots within the receptor-binding domain (RBD) and favour immune evasion [26,31–33]. To gain a deeper insight into the mechanisms of antibody-driven selection processes and, thus, to better predict future developments, it is crucial to investigate the interaction between humoral immune response and infecting virus in immunocompromised patients on a temporal scale and concerning its specificity.

In the case reported here, we analysed the interplay between antibody response and consecutively adapting intra-host virus evolution. Since the patient was closely monitored due to their severely compromised immune system, we were able to study the prolonged SARS-CoV-2 infection and corresponding immune response at high temporal resolution. Our data highlight the importance of the patient’s IgM response as a driver of accelerated viral evolution and its role in viral clearance during the first critical COVID-19 episode. An Omicron infection about one year later was clinically mild and characterised by intense stimulation of IgG and IgA antibodies, primarily targeting cross-reactive epitopes of the first infecting virus strain and the vaccine strain, most likely due to immunological memory and immune imprinting [34, 35]. These results deepen our understanding of escape variant selection in immunocompromised patients and provide methods to monitor the selective pressure exerted by endogenous immune responses in this patient group.

## Materials and methods

### Ethics statement

The patient presented in our study has given informed consent to data collection and publication. The ethics committee of the Aerztekammer Westfalen-Lippe, Muenster, Germany, and the University of Muenster has ethically approved the study *Characterisation of the Humoral Immune Response to SARS-CoV-2 in Vaccinees* under the file number 2021-039-f-S. All study participants provided written informed consent.

### Patient samples

Nasopharyngeal swabs and blood specimens were collected throughout the observation period. The mandatory informed consent declaration about the scientific utilisation of patient material signed by the patient has been archived as stipulated at the University Hospital Muenster. SARS-CoV-2 positive and negative serum pools used as controls were derived from the study *Characterisation of the Humoral Immune Response to SARS-CoV-2 in Vaccinees* approved by the ethics committee of the Aerztekammer Westfalen-Lippe, Muenster, Germany, and the University of Muenster (2021-039-f-S) [36].

### SARS-CoV-2 nucleic acid detection

Viral ribonucleic acid (RNA) was extracted from nasopharyngeal swabs by the *QIAsymphony DSP Virus/Pathogen Kit* (Qiagen N.V., Hilden, Germany) and reverse transcribed using the *SuperScript III Platinum One-Step qRT-PCR System* (Invitrogen/Thermo Fisher Scientific, Waltham, MA, USA) on the *QIAsymphony* instrument (Qiagen). In-house quantitative real-time polymerase chain reaction (qRT-PCR) was performed using the *LightMix Modular SARS-CoV (COVID-19) E-gene* for sarbecovirus envelope (E) gene detection and the *LightMix Modular SARS-CoV-2 (COVID19) RdRP-gene* for diagnostic confirmation of SARS-CoV-2 by RNA-dependent RNA polymerase (RdRP) gene amplification (TIB Molbiol/Roche Diagnostics, Berlin/Mannheim, Germany). According to TIB Molbiol, both kits contain proprietary primer and probe sequences derived from those previously published by Corman and colleagues [37]. Amplification and FAM (5(6)carboxyfluorescein) label detection were performed on a *RotorGeneQ* device (Qiagen) as recommended by TIB Molbiol. Reverse transcription was accomplished at 55°C for 5 min., followed by denaturation at 95°C for 1 min., 40 amplification cycles of 95°C for 5 sec., 60°C for 15 sec., 72°C for 15 sec., and cooling at 40°C for 30 sec.

The viral load in samples was estimated using two quantitative reference samples from INSTAND e.V. (Düsseldorf, Germany) comprising 10,000,000 (Ch07469) and 1,000,000 SARS-CoV-2 RNA genome copies/mL (Ch07470). The mean cycle threshold (Ct) values determined with the reference samples were 22 and 25.

### Whole genome sequencing

Complementary DNA (cDNA) was transcribed from viral RNA isolated from nasopharyngeal swabs using the *LunaScript Reverse Transcription Kit* according to the manufacturer’s instructions (New England Biolabs, Ipswich, USA). Whole genome sequencing (WGS) was performed utilising the *EasySeq SARS-CoV-2 Whole Genome NGS Sequencing Kit* (NimaGen B.V., Nijmegen, The Netherlands) for multiplex amplicon-based preparation of next-generation sequencing (NGS) libraries. Briefly, the amount of cDNA was adjusted to the Ct value as recommended by the manufacturer (NimaGen B.V.) to perform the amplification and barcoding of samples. Subsequently, samples were prepared for running on the *Illumina MiSeq* platform using the 150-base pair (bp) paired-end sequencing chemistry (Illumina Inc., San Diego, CA, USA). All raw sequence data are deposited at the NCBI SRA under bioproject number PRJNA1120916. The resulting fastQ files were further processed (primer removal, quality trimming) and mapped onto the SARS-CoV-2 reference genome NC_045512.3 [38], and variants (substitutions, smaller and larger insertions or deletions) were extracted using the Ridom *SeqSphere+* software version 9 (Ridom GmbH, Muenster, Germany). Variants were filtered based on a variant frequency of at least 1% and a coverage of at least ten-fold.

### Routine serology

Routinely, immunoglobulin G (IgG) antibodies against the nucleocapsid (N) protein (IgG-N) of SARS-CoV-2 were qualitatively assessed by the commercially available, CE/IVD (conformité européenne/in-vitro diagnostic medical devices) certified chemiluminescence microparticle immunoassay (CMIA) *Abbott Architect SARS-CoV-2 IgG* according to the manufacturer’s instructions (Abbott Diagnostics, Abbott Park, North Chicago, Illinois, US). Accordingly, IgG antibodies against the SARS-CoV-2 receptor binding domain (RBD) of the spike (S) protein subunit S1 (IgG-S) were quantified by the CE/IVD certified CMIA *Abbott Architect SARS-CoV-2 IgG II Quant* (Abbott Diagnostics). IgG-S values were expressed as arbitrary units (AU)/mL, values greater than or equal to 50.0 AU/mL indicating seropositivity.

To discriminate between N-, S1- and RBD-specific antibodies, the immunostrip assay *recomLine SARS-CoV-2 IgG* (Mikrogen GmbH, Neuried, Germany, #7374), which contains recombinant target antigens, was performed. The manufacturer claims a sensitivity of 96.3% and a specificity of 98.8%. For assessment of immunoglobulin M (IgM), immunostrips of the IgG kit were probed with IgM-specific reagents derived from the *recom*Line EBV IgM kit (Mikrogen, #4573). Antibody levels were visually determined according to the manufacturer’s guidelines as ordinal values using the cut-off band of immunostrips as an internal reference. Results of individual target-specific bands were rated on an ordinal scale as non-detectable (-), below the cut-off (+/-), with cut-off intensity (+), above the cut-off (++), and very strong intensity (+++).

We applied the CE/IVD certified *cPass SARS-CoV-2 Neutralization Antibody Detection Kit* (GenScript Biotech, Mainz, Germany) to assess the inhibitory capacity of patient antibodies. This surrogate virus neutralisation test (sVNT) quantifies the inhibition of wild-type (wt) RBD binding to human angiotensin-converting enzyme 2 (hACE2) by antibodies in a blocking enzyme-linked immunosorbent assay (ELISA) format and correlates with infectious virus neutralisation assays [39–41]. Following the manufacturer’s manual, patient samples were diluted ten-fold and measured in technical duplicates. Binding inhibition was calculated as 1 - (OD value of sample/OD value of negative control) × 100%. Values less than the cut-off of 30% are considered negative; values at or above the cut-off indicate the presence of SARS-CoV-2 inhibitory antibodies.

### Cloning of spike constructs used in pVNT, sVNT and EIA

Substitution S494P was introduced into the vector pCG1-SARS-2-S-Delta1253, which C-terminally lacks 21 aa (amino acids) as described by Schoefbaenker et al. [36], by opening the plasmid with BamHI and AgeI and inserting PCR fragments amplified from pCG1-SARS-2-S with primer pairs CG1-S-Bam forward (fwd) and CG1-494P reverse (rev), and CG1-494P fwd and CG1-S-Age rev by two-fragment InFusion cloning (Takara Bio, Mountain View, CA, USA). This yielded the vector pCG-SARS-2-S-494P-Delta1253 (**Tables A and B S1 Table**). Accordingly, vectors pCG1-SARS-2-S-484K-494P-Delta1253 and pCG1-SARS-2-S-Delta141-4-484K-494P-Delta1253 containing the substitution E484K and deletion 141–4 were generated by PCR-mediated site-directed mutagenesis and InFusion cloning. Primers and constructs are listed in the **Tables A and B S1 Table**. The spike protein with the Omicron BA.1-specific amino acid sequence and a C-terminal 21aa deletion was expressed from vector pcDNA3.1 SARS-2 Omicron comprising a synthetic S-insert (Thermo Fisher Scientific) as described earlier [36]. All spike sequences were optimised for codon usage.

Cloning of the vectors pEN-secNL-RBD and pEN-secNL-RBD Omicron expressing the secreted form of NanoLuc luciferase (NLuc, Promega, Walldorf, Germany) fused to the wt RBD and Omicron BA.1 RBD, respectively, was delineated by Schoefbaenker et al. [36]. Constructs pEN-secNL-RBD-S494P and pEN-secNL-RBD-484K-494P were generated by opening pEN-secNL-RBD with BamHI and NotI and inserting PCR fragments amplified with primers RBD-BamHI_2 fwd and RBD-NotI rev from pCG1-SARS-2-S-494P-Delta1253 and pCG1-SARS-2-S-484K-494P-Delta1253, respectively, by InFusion cloning (Tables **A and B S1 Table**). The vector pEN-secNL-RBD-E340K was obtained by opening pEN-secNL-RBD with NheI and NotI and inserting PCR products amplified from pEN-secNL-RBD with primer pairs secNL fwd and wt E340K rev, and wt E340K fwd and RBD Not1 rev by two-fragment InFusion cloning. The construct pEN-secNL-RBD-Omicron-E340K was generated in the same way using pEN-secNL-RBD Omicron as the target sequence and primers Omicron RBD E340K fwd and rev to introduce the substitution E340K (Tables **A and B S1 Table**).

Plasmid pEN-secNL-15-307 expressing the N-terminal domain (NTD) of SARS-CoV-2 S fused to secreted NanoLuc luciferase (secNL) was generated as described by Schoefbaenker et al. [36]. Vectors pEN-secNL-NTD-Delta141-4 and pEN-secNL-NTD-Omicron were generated accordingly by inserting PCR products amplified with primers S15-BamH1 fwd and S307-Not1 rev from pCG-1-SARS-2-S-Delta141-4-484K-494P-Delta1253 and pcDNA3.1 SARS-2 Omicron, respectively.

### Pseudovirus-based virus neutralisation test

SARS-CoV-2 neutralisation was analysed with the SARS-CoV-2 S protein-bearing green fluorescent protein (GFP)-expressing vesicular stomatitis virus (VSV) pseudotyped system (VSV-pVNT) as described earlier [36, 42, 43]. As depicted by Mohamed and colleagues, the assay quantifies the inhibition of cell entry by VSV particles exhibiting SARS-CoV-2 spike proteins on their surface by neutralising antibodies [42]. Vectors used to express wt S and variant forms of S are listed in the **Table B in S1 Table**. Patient sera were tested in the pVNT at 1:20, 1:80, and 1:320 dilutions. Four technical replicates were performed, and the mean was calculated. SARS-CoV-2 S antibody negative and positive sera pools derived from samples acquired in the context of a vaccination study as described by Schoefbaenker and colleagues [36] were used as controls. GFP-positive cells were quantified with the Celigo Image Cytometer (Nexcelom/Perkin Elmer Inc., Waltham, MA, USA). The degree of neutralisation was calculated as the reduction of the GFP signal (%) = (1 –GFP-positive cells of the treated sample/GFP-positive cells of the untreated sample) x 100%. The reduction of GFP-positive cells by ≥50% was rated as a positive result in the pVNT [36]. The 50% inhibition values (IC50) were calculated using best-fit curves.

### In-house sVNT and in-house immunoglobulin capture EIA

Expression of the secreted NanoLuc luciferase (NLuc)-tagged recombinant proteins described above by transient transfection of cell cultures and quantification of inhibitory antibodies by the in-house sVNT and immunoglobulin capture enzyme-linked immunoassay (EIA) were performed as described [36]. Sera were tested in the sVNT at a dilution of 1:20. Luciferase activity in the absence of human serum, and SARS-CoV-2 antibody negative and positive human serum pools outlined above served as controls. Inhibition of the binding of the RBD to hACE2 was calculated as a reduction of the NLuc signal (%) = (1 –NLuc signal of the sample/NLuc signal of the untreated sample) x 100. The cut-off was set at 25% reduction.

To determine IgG, immunoglobulin A (IgA), and IgM antibodies in human sera using secNLuc-tagged spike antigens in a heavy chain-capture EIA, sera were tested at a dilution of 1:100. Values were expressed as Ig-class-specific activity (rlu) subtracted by activity in the absence of serum samples. The SARS-CoV-2 positive and negative serum pools mentioned above served as controls. The cut-off values of the RBD-specific and the NTD-specific EIA were set at 300 rlu and 100 rlu, respectively.

## Results

### Patient history

We report a patient in their 60s diagnosed with acute myeloid leukaemia (AML M1, intermediate risk by ELN). They initially received chemotherapy according to the 7+3 regimen plus gemtuzumab ozogamicin, followed by therapy with high-dose cytarabine/gemtuzumab ozogamicin. Due to AML relapse six months later, the first allogeneic stem cell transplant was performed. After ten months, the patient suffered another AML relapse, whereupon a second allogeneic stem cell transplant was performed. As a result, the patient developed graft-versus-host disease in the intestine (grade III), liver (grade II), and skin (grade II), prompting continuous therapy with ciclosporin A in serum level-adapted doses. Six months after transplantation, Epstein-Barr virus-induced lymphoproliferation was detected, necessitating B-cell depleting therapy with rituximab.

### The clinical course of SARS-CoV-2 infection

**Fig 1:**
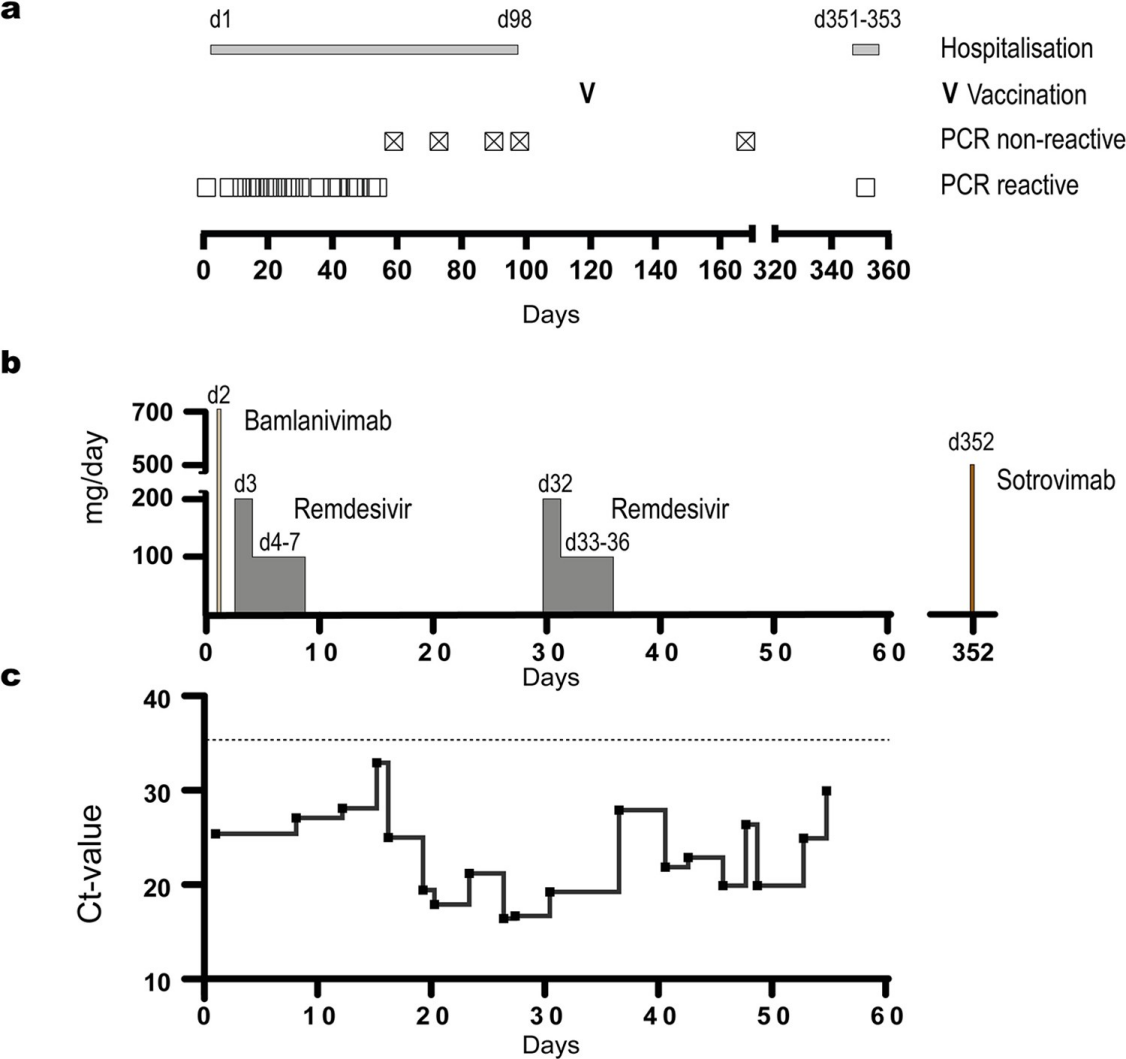
Course of the first and second episode of SARS-CoV-2 infection. **(a)** The figure displays the duration of hospitalisation following the initial diagnosis (day 1) (grey bar), the time point of the first vaccination (V), and the detection of SARS-CoV-2 RNA in nasopharyngeal swabs by qRT-PCR. Positive and negative PCR results are indicated by squares and crossed-out squares, respectively. The day of sampling is indicated on the x-axis. **(b)** The graph illustrates the time course of the administration and dosage of antiviral therapeutics received by the patient, with the timeline indicated on the x-axis. **(c)** The relative viral load in nasopharyngeal swabs during the first episode is indicated by Ct values of qRT-PCR; the timeline of sampling is delineated on the x-axis.

When the patient was admitted to the hospital two months after rituximab therapy for *Legionella* pneumonia and consecutive cardiac decompensation, the AML was in remission, hence no oncological medication was administered. During hospitalisation, the patient developed coughs and progressive dyspnoea. SARS-CoV-2 infection was confirmed by qRT-PCR in a nasopharyngeal swab (defined as day 1 of the disease) (**Fig 1A**). On admission to the isolation ward, laboratory analysis revealed leukopenia (2.420/μL) with 90.4% neutrophils and marked lymphopenia (1.7% lymphocytes). Immunoglobulins were markedly decreased (IgG 458 mg/dL), and CD19+ B lymphocytes were almost completely depleted at 0.2% (1 cell/μL absolute). CD4+ T lymphocytes (25.6%, 81 cells/μL) were significantly reduced, while CD8+ T lymphocytes were within the normal range at 85.3%. The development of the B cell and CD3+, CD4+ and CD8+ T cell populations and the serum levels of immunoglobulins G, M, and A are summarised in the **Tables A-E in S2 Table**.

Following the COVID-19 treatment guidelines at the time, the patient received monotherapy with the S-specific monoclonal antibody bamlanivimab (700 mg) on day 2. In addition, they were treated with remdesivir for five days (200 mg on day 3 and 100 mg on days 4 to 7) (**Fig 1B**). Upon initiation of treatment, dyspnoea declined, whereas fatigue and intermittent diarrhoea persisted. On day 19 fever and coughs reoccurred. Again, remdesivir was administered for five days (days 32 to 36). The patient tested negative for SARS-CoV-2 on days 56 and 57. The first vaccination against SARS-CoV-2 was carried out with Spikevax (mRNA 1273, Moderna) on day 122. On day 351, the patient experienced a second episode of COVID-19 and received sotrovimab (500mg) on day 352 (**Fig 1B**). Re-infection resulted in a rapidly self-limiting disease associated with mild symptoms, e.g., headache, myalgia, rhinorrhoea and loss of taste. The patient was monitored in short intervals for the presence of SARS-CoV-2 RNA in nasopharyngeal swabs by qRT-PCR. On day 1, a cycle threshold (Ct) value of approx. 25 was determined, which corresponds to a viral load of roughly 10^6^ copies of SARS-CoV-2 RNA/mL. Upon commencement of bamlanivimab and remdesivir treatment, RNA levels decreased during the first two weeks. Viral load in swabs reached levels close to the detection limit of PCR on day 15 (**Fig 1C**). Starting on day 16, viral load in swabs strongly increased and peaked around days 26 and 27. The second remdesivir therapy was associated with a transient reduction of viral RNA levels between days 30 and 36. After completion of remdesivir treatment, the second rise in viral RNA levels was observed, peaking on days 45 and 48. At later time points, RNA levels of nasopharyngeal swabs strongly decreased, and the last positive sample was acquired on day 54 (**Fig 1C**). In swabs collected between days 73 and 168 viral RNA was not detectable. On day 351, SARS-CoV-2 re-infection was diagnosed by two independent point-of-care tests, i.e., ID NOW COVID-19 (Abbott) and BioFire Respiratory Panel 2.1 *plus* (bioMérieux, Nürtingen, Germany). High levels of SARS-CoV-2 RNA (Ct value 13.14) were found in a nasopharyngeal swab on day 352 (**Fig 1A**).

### Sequencing results

**Fig 2:**
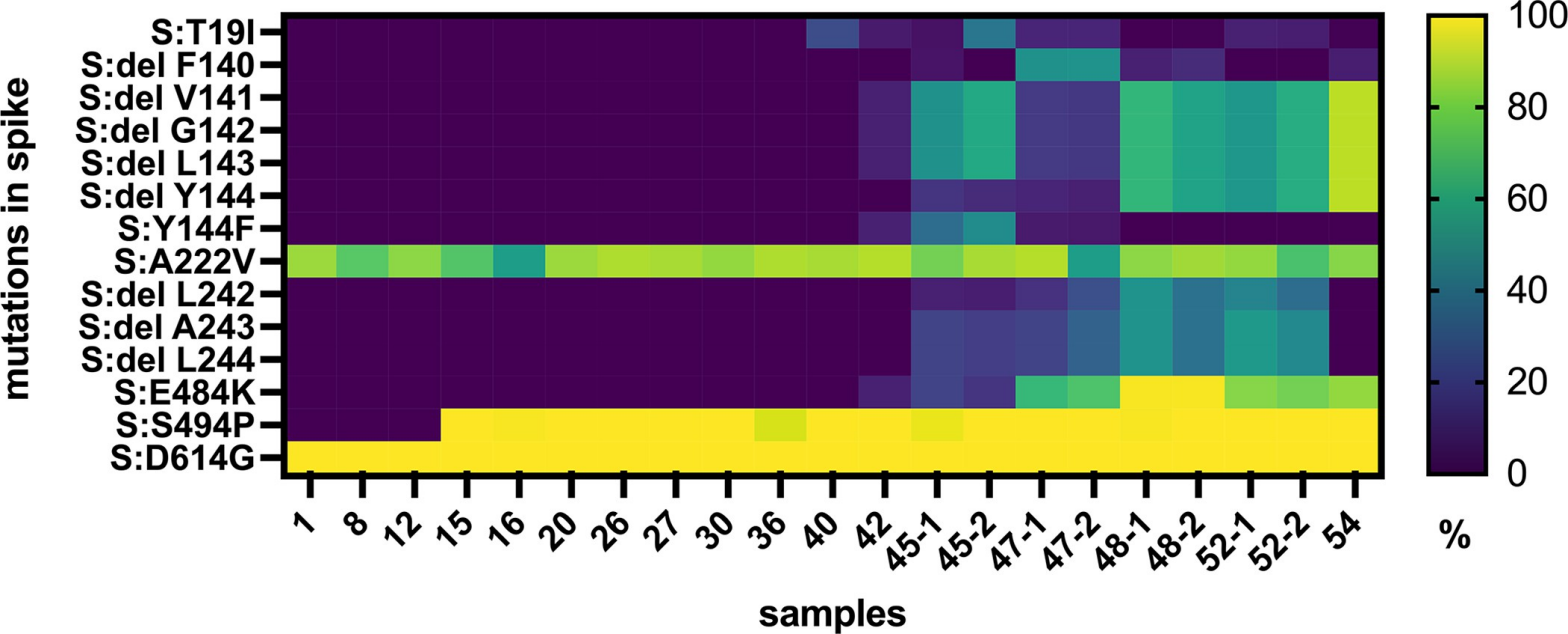
Intra-host evolution of SARS-CoV-2 during the first episode of infection. The relative frequency of mutations in spike (% of reference sequence) is marked with a colour scale as indicated. The day of sampling after the initial diagnosis (day 1) and the days on which two consecutive samples were sequenced are indicated. GISAID accession numbers of the consensus sequences are provided in the **S3 Table**.

The significant increase in RNA levels, starting 16 days after disease onset, indicated virological failure of the combined bamlanivimab and remdesivir therapy. This prompted us to search for escape mutations by whole genome sequencing of viral RNA from nasopharyngeal swabs. Sequence analysis on day 1 revealed that the patient had initially been infected with SARS-CoV-2 of PANGOLIN lineage B.1.177.81 (20E, EU1) [44], a variant that became rapidly dominant in Europe during the summer of 2020 [45]. Within the S gene typical amino acid (aa) exchanges of this lineage were found (S:A222V, S:D614G) (**Fig 2 and S3 and S4 Tables**). On day 15, i.e., shortly before the first relapse, the exchange S:S494P within the RBD of the S protein was revealed (**Fig 2 and S3 and S4 Tables**). Additional substitutions in the S protein were not observed. Variant virus carrying solely the S:S494P substitution dominated from days 15 to 40.

Coinciding with the second, biphasic peak in viral RNA levels, the phase of apparent genomic stability of the variant S:S494P ended and several additional mutations emerged in short succession within the NTD and the RBD of S until virus elimination on day 56. The S:T19I substitution was detected with varying frequency on days 40, 42, 45, 47 and 52 (**Fig 2 and S3 and S4 Tables**). Between days 42 and 54, several substitutions and deletions appeared in the NTD. Within the repeated deletion region (RDR) 2 of the NTD, the S:Delta141-3 Y144F mutation was first detected on day 42 and reached its highest frequency on day 45. This mutation was replaced by S:DeltaF140 on day 47, which in turn was followed by S:Delta141-4, reaching almost 90% frequency on day 54. The S:Delta242-4 mutation in the RDR4 of the NTD was detected from days 48 to 52 and reached its highest frequency on days 48 and 52. Within the RBD, the S:E484K substitution was first detected on day 42, persisted until virus elimination, reached high frequency between days 47 and 54 and peaked on day 48 (**Fig 2 and S3 and S4 Tables**). Two samples were obtained and sequenced in parallel on days 45, 47, 48 and 52. Overall, the results obtained from samples collected on the same day were consistent, but differences in mutation frequencies indicated dynamic changes in the composition of viral variants (**Fig 2 and S4 Table**).

Sequencing of the SARS-CoV-2 RNA from the swab obtained on day 352 confirmed re-infection with the SARS-CoV-2 variant Omicron BA.1.18 (**S3 Table**).

### Humoral immune response

Close monitoring of antibody reactivity in peripheral blood samples enabled a detailed analysis of the humoral immune response to both infection episodes. A total of 17 serum samples were available for serological testing. Eight samples were obtained during the first episode of infection (days 2, 8, 17, 32, 40, 45, 49, and 56). Two sera were collected after virus elimination (days 67 and 90), and two sera were taken again after the first vaccination (days 131 and 138). Finally, five sera were obtained before (day 326), during (days 352 and 353) and after re-infection with Omicron BA.1.18 (days 381 and 391).

### SARS-CoV-2 wild-type-reactive binding antibodies

**Fig 3:**
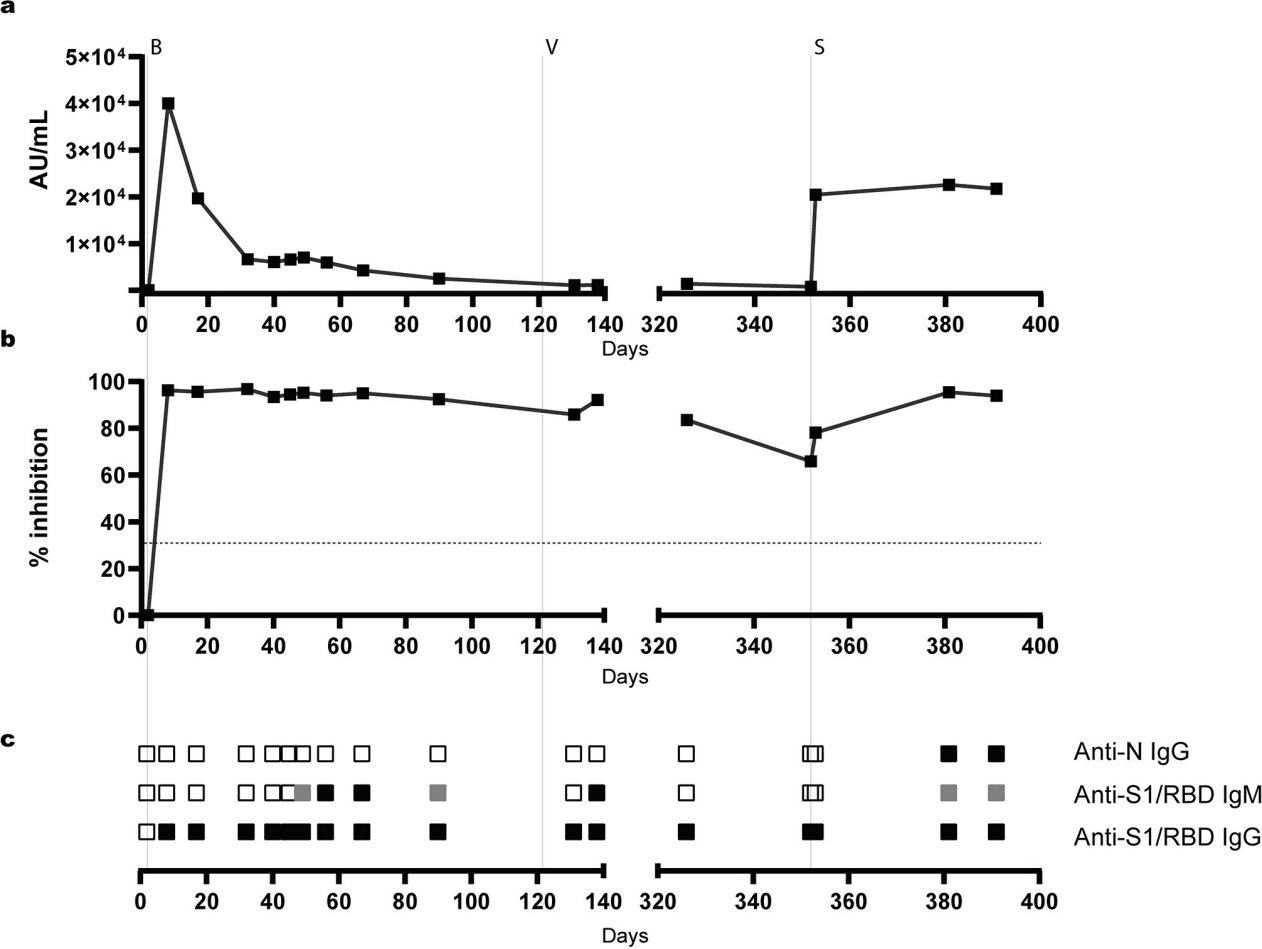
Detection of SARS-CoV-2 wild-type-reactive binding antibodies by routine serologic assays. Peripheral blood samples obtained from the patient on the days indicated were tested by the quantitative Abbott Anti-RBD-CMIA **(a)** and the GenScript cPass sVNT **(b)**. Antibody levels detected by Anti-RBD-CMIA and sVNT are given as arbitrary units (AU)/mL, cut-off ≥50 AU/mL, and signal reduction (% inhibition, cut-off >30%) as compared to the negative control. The GenScript cPass sVNT was performed with technical duplicates at a serum dilution of 1:20. The cut-off of the sVNT is indicated by the horizontal dotted line. **(c)** Qualitative results of the Mikrogen line blot testing for Anti-N IgG, as well as Anti-S1 and Anti-RBD IgM and IgG, are indicated as follows: open squares: non-reactive, grey squares: reactive below cut-off, black squares: reactive above cut-off. Administration of bamlanivimab (B), first vaccination (V) and therapy with sotrovimab (S) is indicated by vertical dotted lines.

In the quantitative, RBD-specific SARS-CoV-2 CMIA (Abbott), IgG antibodies were absent in the first blood sample obtained on day 2, reached their highest levels on day 8 after administration of bamlanivimab, and decreased exponentially afterwards, consistent with the mean apparent terminal elimination half-life of approximately 17 days published for bamlanivimab [46]. The first vaccination on day 122 did not significantly increase antibody levels on days 131 and 138 (**Fig 3A**). When re-infection with the Omicron BA.1 variant was diagnosed on day 352, antibody levels were slightly lower as compared to day 138. Administration of 500 mg sotrovimab on day 353 resulted in a strong increase in RBD-specific IgG antibodies. Comparably high levels of RBD-specific IgG levels were observed on days 381 and 391. IgG antibodies against the nucleocapsid (N) protein were not detected in CMIA (Abbott) until day 381 (**S5 Table**).

The commercial surrogate virus neutralisation test cPass (GenScript Biotech) was used to quantify levels of antibodies inhibiting the binding of the RBD to ACE2 [41, 47]. As seen in the quantitative S EIA and line blot, the patient tested negative on day 2. Administration of bamlanivimab resulted in high levels of inhibitory antibodies, which slowly decreased until day 131. The effect of vaccination was reflected by a small increase in inhibitory antibodies on day 138 (**Fig 3B**). Administration of sotrovimab on day 352 resulted in a moderate increase in antibody levels in sVNT, whereas high levels of inhibitory antibodies were detected on days 381 and 391.

Qualitative analysis of the IgG response by the Mikrogen line blot assay confirmed the results of the CMIA. SARS-CoV-2-specific antibodies were absent before the administration of bamlanivimab, whereas strong IgG reactivity with S1 and RBD was observed in all samples obtained at later times. N-specific IgG antibodies were only detected on days 381 and 391. Determination of SARS-2-specific IgM antibodies by line blot indicated the presence of IgM antibodies against S1 and RBD on days 49 to 90, day 138 and days 381 and 391 (**Fig 3C**).

Overall, the results of routine serology suggested the onset of an endogenous humoral immune response at the end of the first infection period and immune responses to vaccination and re-infection with the Omicron variant. This prompted us to better characterise the patient´s humoral immune response in various S-specific in-house assays with an emphasis on neutralising and/or variant-specific antibodies.

### Variant-specific neutralising antibodies

**Fig 4:**
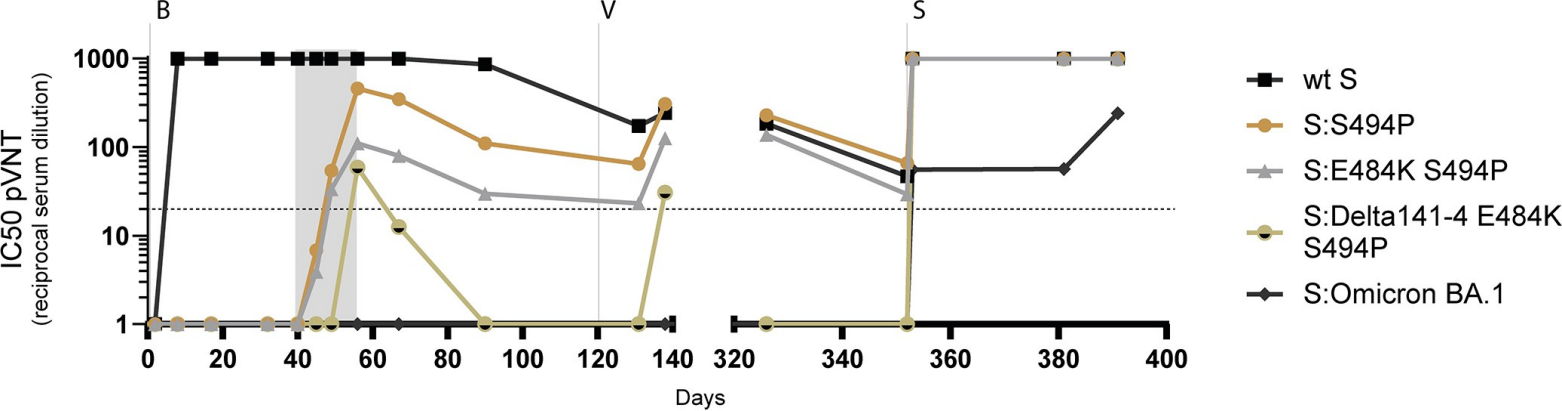
Detection of variant-specific neutralising antibodies. The reactivity of the patient’s sera with wt S, S:S494P, S:E484K S494P, S:Delta141-4 E484K S494P and S Omicron BA.1 was determined by pVNT. The mean inhibition of the GFP signal (% reduction as compared to the untreated control) was determined for four technical replicates at a serum dilution of 1:20, 1:80 and 1:320. Inhibition by ≥50% at a dilution of 1:20 was rated as a positive result. The half-maximal inhibitory activity of serum samples (IC50) was calculated using best-fit curves. The IC50 values are presented as reciprocal serum dilutions. IC50 values exceeding 1000 or below 1 are depicted as 1000 or 1, respectively. The interval between the first detection of additional mutations in the NTD and RBD in variant S:S494P on day 40 and virus elimination on day 56 is indicated by grey shading. The administration of bamlanivimab (B), the first vaccination (V) and sotrovimab (S) therapy are indicated by vertical dotted lines. The horizontal dotted line represents the cut-off value for the lowest serum dilution tested (1: 20). The inhibition of wt S and S variants at individual serum dilutions is depicted in the **S1 Fig**.

Levels of neutralising antibodies against wt S and S variants were quantified by VSV pVNT in blood samples serially diluted 1:20, 1:80, and 1:320. During the first episode of infection, S-specific neutralising antibodies were not detected before administration of bamlanivimab, which resulted in high neutralising antibody titres (IC50 ≥1:1000) against wt S. Neutralising antibodies reactive with variants S:S494P, S:E484K S494P, and S:Delta141-4 E484K S494P started to rise on day 45 and reached 50% inhibition (IC50) at a serum dilution of 1:20 or higher on days 49 (S:S494P, S:E484K S494P) and 56 (S:Delta141-4 E484K S494P) (**Figs 4 and S1**). As compared to wt S-specific antibodies, levels of these variant-reactive antibodies were significantly lower, peaked on day 56, and then decreased again. On day 56, IC50 titres of neutralising antibodies against S:S494P were approximately 4-fold higher as compared to S:E484K S494P and approximately 8-fold higher than those against S:Delta141-4 E484K S494P. Neutralising antibodies against S Omicron (BA.1) were not detected during the first episode of SARS-CoV-2 infection (**Figs 4 and S1**).

To analyse the immune evasion properties of the S variants observed, an additional test was conducted to determine the neutralisation capacity of nine sera obtained from healthy vaccinees. The S:Delta141-4 E484K S494P mutant was found to evade neutralisation by sera of healthy controls obtained four weeks after their first vaccination to a highly significant extent (**S2 Fig**). A comparable immune escape was observed with S:Omicron BA.1, yet not with S:S494P and S:E484K S494P. After the second vaccination, immune evasion was no longer detected in the case of S:Delta141-4 E484K S494P. However, it persisted in the case of S:Omicron BA.1.

The administration of the first vaccine dose on day 122 resulted in increased antibody levels against wt S, S:S494P, S:E484K S494P, and S:Delta141-4 E484K S494P on day 138. Neutralising antibodies reactive with S Omicron BA.1 were not elicited. Approximately 4 weeks before (day 326) and immediately upon the onset of the second infection episode (day 352), neutralising antibodies against wt S and variants S:S494P and S:E484K S494P were detected, whereas no reactivity was found with S:Delta141-4 E484K S494P and S Omicron BA.1 (**Figs 4 and S1).** The administration of sotrovimab on day 353 led to strong reactivity with wt S and all variants tested including S Omicron, albeit the increase in S Omicron BA.1-reactive neutralising antibody titres was significantly lower. During the follow-up period neutralising antibodies against S Omicron BA.1 increased from day 381 to day 391. The levels of neutralising antibodies against wt S, S:S494P, S:E484K S494P, and S:Delta141-4 E484K S494P remained unchanged.

### Variant-specific inhibitory antibodies

**Fig 5:**
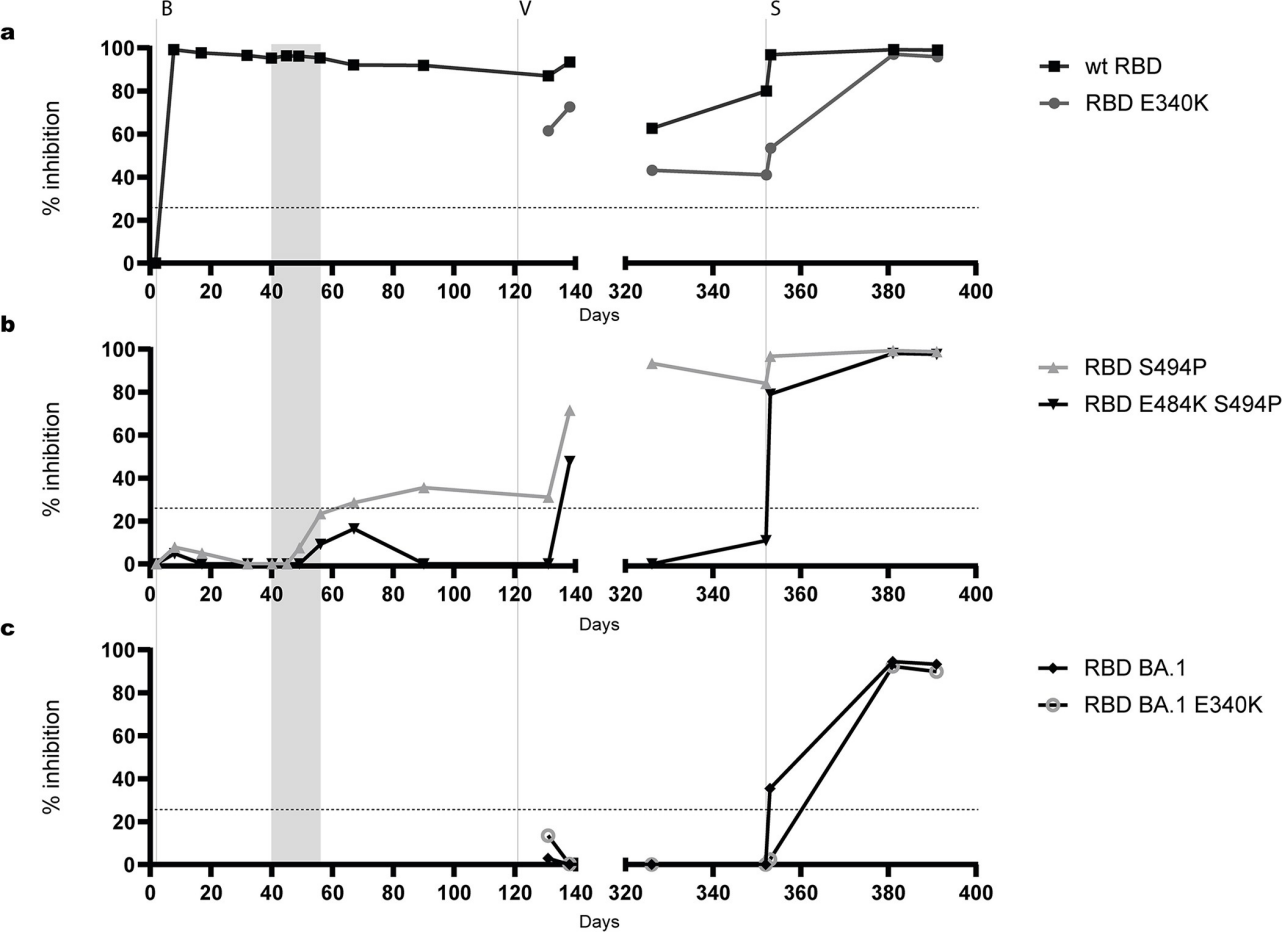
Detection of variant-specific inhibitory antibodies. The reactivity of patient sera with wt RBD and RBD E340K **(a)**, RBD S494P and RBD E484K S494P **(b)**, and RBD Omicron BA.1 and RBD Omicron BA.1 E340K **(c)** was determined by sVNT using N-terminally secNLuc-tagged RBD antigens. Sera were tested at a dilution of 1:20. The mean values of two independent experiments are shown. The cut-off value of the sVNT for wt RBD was set to ≥25% inhibition (dotted line). Values below 0% were set to zero. The period between the first detection of additional mutations in the NTD and RBD in variant S:S494P on day 40 and virus elimination on day 56 is shaded in grey. The day of sampling after the initial diagnosis (day 1) is indicated on the x-axis. The administration of bamlanivimab (B), the first vaccination (V) and sotrovimab (S) therapy are indicated by vertical dotted lines.

RBD-fragments N-terminally tagged with secNLuc were used as antigens in the in-house sVNT to detect variant-specific antibodies inhibiting binding to ACE2. The course of inhibitory antibodies directed against wt RBD closely followed the results obtained in the cPass sVNT (**Fig 3B**). This showed that the administration of bamlanivimab resulted in high levels of inhibitory antibodies, which slowly declined during the first episode of infection. Vaccination moderately increased antibody titres against wt RBD. Wt RBD-specific inhibitory antibodies were also detected on day 326 before Omicron re-infection, increased on day 353 due to the administration of sotrovimab, and remained at high levels on days 381 and 391 (**Figs 5A and S3**).

The binding of RBD S494P and RBD E484K S494P to ACE2 was not significantly inhibited by the administration of bamlanivimab, highlighting the specificity of the in-house sVNT. An increase in inhibitory antibodies against RBD S494P was observed from days 49 to 56. Antibody levels above the cut-off of the in-house sVNT defined for wt RBD in a vaccination study, i.e., 25% inhibition as described by Schoefbaenker et al. [36], were first detected on day 67 and remained above the cut-off level in all serum samples taken at later time points. Inhibitory antibodies against RBD E484K S494P did not reach the cut-off of 25% inhibition during the first episode but showed an increase between days 56 and 67 (**Figs 5B and S3**).

Vaccination increased inhibitory antibody levels against RBD S494P and to a lesser extent against RBD E484K S494P. On day 326, before re-infection with Omicron, inhibitory antibodies against RBD E484K S494P were not detected. Administration of sotrovimab resulted in a strong increase in sVNT titres against RBD S494P and RBD E484K S494 (**Figs 5B and S3**). The presence of inhibitory antibodies reactive with RBD Omicron BA.1 was determined in response to vaccination and re-infection. RBD Omicron BA.1-reactive inhibitory antibodies were not detected in sVNT until the administration of sotrovimab, which only moderately increased antibody levels. On days 381 and 391, high levels of RBD Omicron BA.1-reactive antibodies were found, indicating the boosting of inhibitory antibodies by the Omicron re-infection (**Figs 5C and S3**).

To reduce the effect of sotrovimab on the reactivity of serum samples in the sVNT, the substitution S:E340K associated with high-level resistance against sotrovimab was introduced into wt RBD and RBD Omicron BA.1 [48, 49]. In contrast to RBD constructs lacking E340K, administration of sotrovimab on day 353 had only a minor or no effect on antibody reactivity with RBD E340K and RBD Omicron E340K in sVNT (**Figs 5A, 5C and S3**). Subsequently, a strong increase of inhibitory antibodies against RBD 340K and RBD Omicron BA.1 E340K between days 353 and 381 was observed, which further corroborates the triggering of an inhibitory antibody response by Omicron re-infection.

### Variant-specific RBD- and NTD-binding antibodies

**Fig 6:**
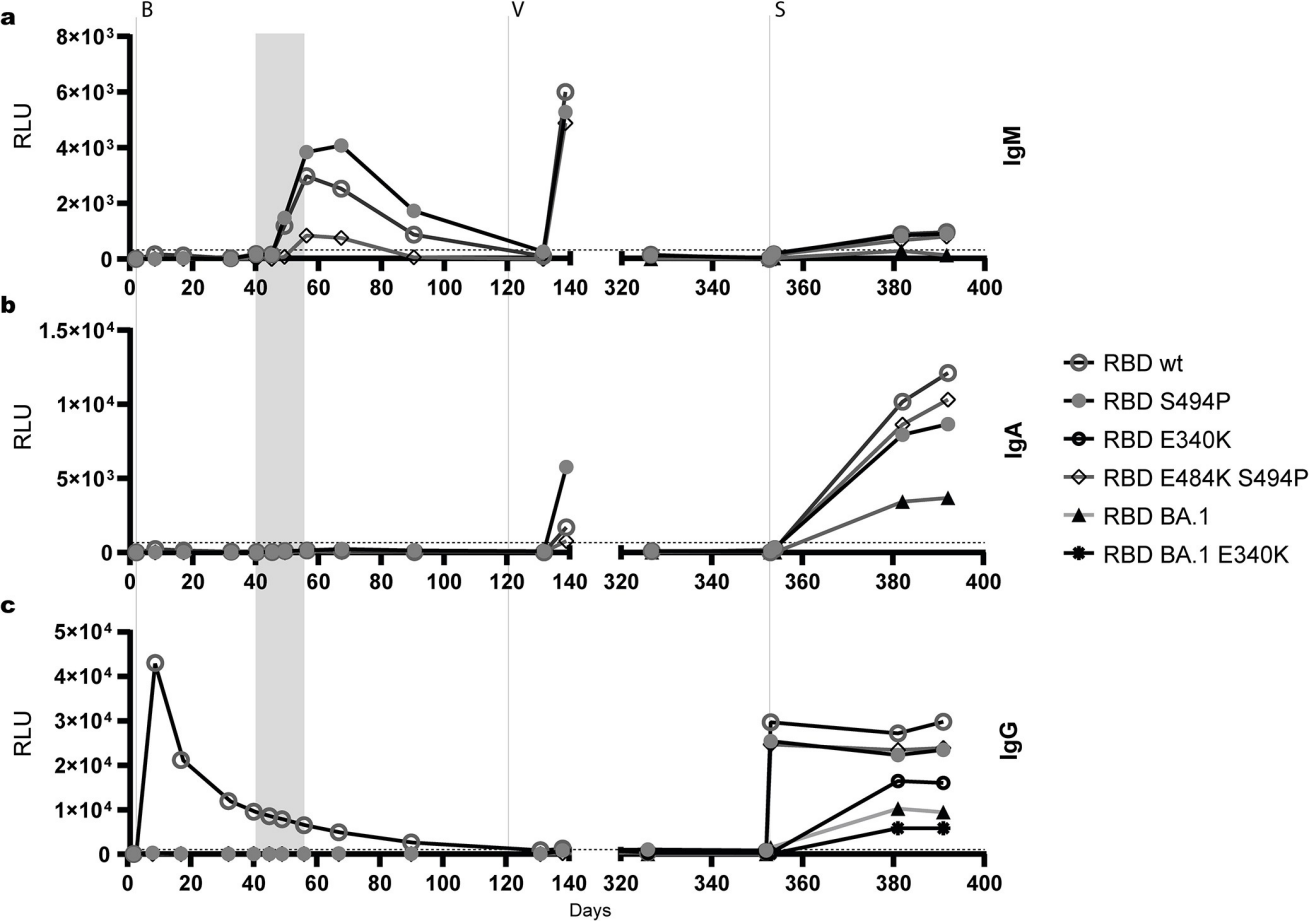
Variant-specific serum reactivity in the Ig class capture RBD-EIA. IgM reactivity **(a)**, IgA reactivity **(b)**, and IgG reactivity **(c)** of the patient’s sera. Antibody reactivity against wt RBD, RBD S494P, and RBD E484K S494P was determined from days 2 to 391, and reactivity against RBD Omicron BA.1 from days 131 to 391. Additionally, IgG reactivity with RBD E340K and RBD Omicron BA.1 E340K was tested from days 131 to 391 **(c)**. Sera were analysed at a dilution of 1:100. Mean values of two independent experiments are shown. The cut-off was set at 300 rlu. Signal strength is given in relative light units (rlu). Values below 0 rlu were set to zero. The period between the first detection of additional mutations in the NTD and RBD in variant S:S494P on day 40 and virus elimination on day 56 is shaded in grey. The day of sampling after the initial diagnosis is indicated on the x-axis. The administration of bamlanivimab (B), the first vaccination (V) and sotrovimab (S) therapy are indicated by vertical dotted lines.

To delineate the pattern of RBD-specific IgM, IgA, and IgG antibody responses, secNLuc-tagged RBD-fragments were additionally applied as diagnostic antigens in an in-house Ig class capture EIA. During the first episode of infection, a prominent IgM response against wt RBD, RBD S494P, and RBD E484K S494P peaking around days 56 to 67 was detected. The strongest IgM signal in EIA was observed with wt RBD and RBD S494P, reactivity with RBD E484K S494P was lower. In contrast, RBD-specific IgA responses or an IgG response against RBD S494P and RBD E484K S494P were not observed. As expected, administration of bamlanivimab resulted in very high IgG reactivity with wt RBD on day 8 followed by exponentially decreasing antibody levels between day 8 and 90 (**Figs 6A–6C and S4**). The initial vaccination resulted in the production of IgM and IgA antibodies reactive with wt RBD, RBD S494P, and RBD E484K S494P. However, this did not lead to a significant increase in RBD-specific IgG antibodies. IgM, IgA, and IgG antibodies reactive with RBD Omicron BA.1 were not induced by vaccination (**Figs 6A–6C and S4**).

In contrast to the first infection, re-infection with the Omicron variant resulted in low IgM and strong IgA responses, which were dominated by antibodies reactive with wt RBD, RBD S494P, and RBD E484K S494P, however, also contained lower levels of antibodies reactive with RBD Omicron. The prominent IgG reactivity with wt RBD, RBD S494P, and RBD E484K S494P on days 353 to 391 appeared to be mainly caused by administration of sotrovimab. The effect of sotrovimab was much less pronounced on IgG reactivity with RBD Omicron BA.1. A rise in IgG reactivity with RBD Omicron BA.1 from day 353 to 381 indicated the onset of an endogenous RBD-specific IgG response. This was confirmed using the sotrovimab escape-mutants RBD E340K and RBD Omicron E340K as diagnostic antigens. With both antigens IgG levels increasing from day 353 to 381 were observed (**Figs 6A–6C and S4**).

**Fig 7:**
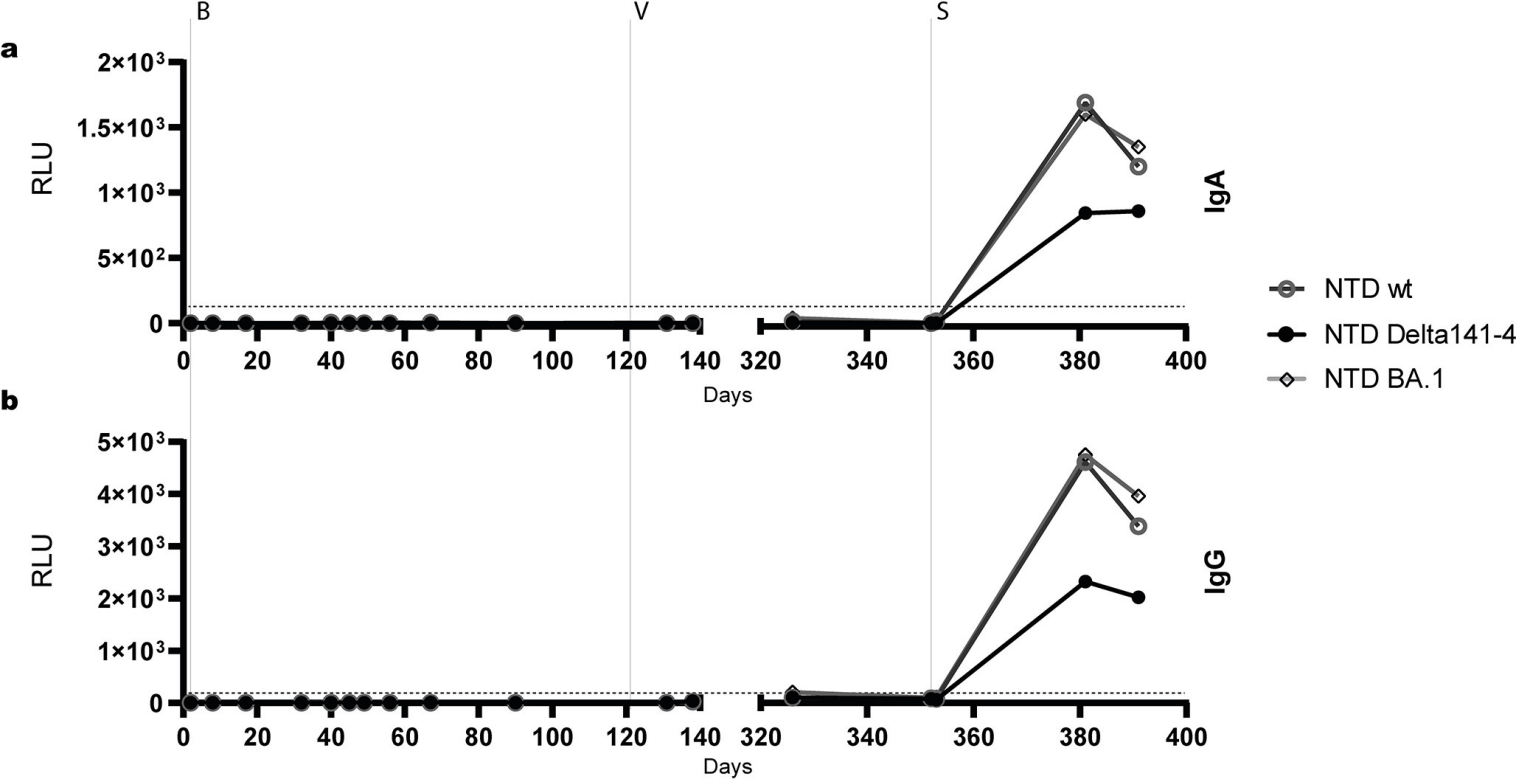
Variant-specific serum reactivity in the Ig class capture NTD-EIA. **(a)** IgA reactivity, **(b)** IgG reactivity. Sera were tested at a dilution of 1:100. The means of two independent experiments are shown. Antibody reactivity is given in relative light units (rlu). Values below 0 rlu were set to zero. The cut-off was set at ≥ 100 rlu. The day of sampling after the initial diagnosis is indicated on the x-axis. The administration of bamlanivimab (B), the first vaccination (V) and sotrovimab (S) therapy are indicated by vertical dotted lines.

Finally, antibody reactivity with secNLuc-tagged NTD fragments of wt S and variants S:E484K-S494P-Delta141-4 and S Omicron BA.1 was tested in the Ig class capture EIA (**Figs 7A, 7B and S4**). Neither during the first episode of infection nor after the first vaccination were significant levels of NTD-specific IgG, IgM, and IgA antibodies detected. In contrast, re-infection with Omicron induced reactivity of NTD-specific IgG and IgA antibodies on days 381 and 391. Levels of IgG antibodies against wt NTD and NTD-Omicron were higher as compared to antibodies against NTD-Delta141-4. Prominent IgM responses against the NTD were still not observed both in the patient sera and with the positive control (**S4 Fig**).

## Discussion

Viral rebound during prolonged infection has been identified to be strongly associated with antibody evasion [13]. In the case described here, two phases of viral rebound were observed. The first viral rebound was caused by the emergence of the known bamlanivimab escape mutation S:S494P [50,51]. The substitution S:S494P emerged between days 12 and 15, reaching a frequency of almost 100% already by day 15 and persisting in subsequent samples. Given the half-life of bamlanivimab, which is approximately 17 days [46], it can be assumed that bamlanivimab monotherapy early in infection imparted potent neutralisation of the parental virus throughout the first episode of infection. This was confirmed by the detection of high levels of neutralising antibodies against wt S until viral clearance on day 56 and beyond. No substitutions at other positions associated with bamlanivimab escape, such as L452, E484, G485, F490 and Q493 [52–57], have been identified during the initial six weeks of infection.

The second viral rebound occurred concurrently with the emergence of variants harbouring various additional mutations within spike between days 40 and 48. Of note, variants carrying substitutions and deletions within the NTD at positions T19, F140 to Y144, and L242 to L244, as well as the substitution E484K within the receptor binding motif (RBM) appeared. Of these, the triple mutant S:Delta141-4 E484K S494P became stably established and reached fixation until virus elimination.

Substitutions at position 484, such as E484K, are present in a range of variants including former variants of concern (VOCs) and variants of interest (VOIs). Located in an epitope recognised by most individuals with anamnestic infection and vaccination, they confer partial resistance to convalescent sera and broad resistance to bamlanivimab and other class 2 neutralising monoclonal antibodies [48,52,53,55–57]. Accordingly, the emergence of E484 substitutions was frequently observed in patients with prolonged infection. Bamlanivimab treatment [9,54,58–63] and the administration of polyclonal anti-S-positive immunoglobulin preparations such as CCP [13,54,60,62,64–67] have been reported to give rise to these escape mutations. In other instances, E484 substitutions were detected in the absence of passively transferred anti-S-antibodies, suggesting the positive selection of escape mutants by endogenous antibody responses [8,13,68–71].

Several authors have reported the appearance of single substitutions at positions Q493 or S494 [61,72–77] and double substitutions at positions E484 and Q493/S494 [9,58,63,64,70] during prolonged infection, albeit less frequently than E484 substitutions. In the majority of these cases, antibody-based therapies may have contributed to the selection of these double mutants [9,58,63,64]. Alenquer and colleagues have shown an increased escape of mutant S:E484K S494P from neutralisation by convalescent sera [51]. In several recent Omicron subvariants, the substitution S:S494P occurs simultaneously with substitutions at position E484, apparently conferring growth advantages [33]. Thus, it can be concluded that in the case reported here, the combination of substitutions S:E484K and S:S494P may have synergistically enhanced antibody evasion. Accordingly, we did not observe a reversion of S:S494P to the wt-like haplotype upon the emergence of S:E484K. In addition, the strong selective pressure exerted by bamlanivimab early in infection may have caused the replacement of the parental wt-like virus by the outgrowing resistant variant S:S494P.

Within the NTD, the substitution T19I was first detected on day 40 and persisted until day 52. In mutant S:D614G, this substitution has been demonstrated to slightly reduce the neutralising activity of RBD-specific class I monoclonal antibodies and some NTD-specific monoclonal antibodies [78]. In addition, T19I has been identified in certain Omicron lineages [79]. Therefore, substitution T19I may have contributed to the antibody escape observed in the case reported here.

The emergence of substitutions and deletions in the repeated deletion regions RDR2 and RDR4 of the NTD, which are known to interfere with the binding of antibodies to the so-called NTD supersite [53,80–84], was first detected on day 42 of infection in our patient, i.e., simultaneously with substitution E484K. Notably, substitutions and deletions similar to those identified in our patient, such as S:Delta140, S:Delta141-3 Y144F, S:Delta141-4 and S:Delta242-4, are also present in the former VOCs B.1.1.7 (Alpha) (S:Delta69-70 S:Delta144), B.1.351 (Beta) (S:Delta242-4), and B.1.1.529 (Omicron) (S:Delta143-5) [81]. The emergence of deletions in the NTD supersite, which belong to the escape mutations most frequently found during prolonged infection, has been documented in numerous case reports and clinical observational studies. In multiple studies, NTD deletions were observed to occur in temporal relation to ABT based on polyclonal antibodies [13,54,62,64,66,71,73,84–91]. Furthermore, deletions in the NTD supersite have been reported to emerge in the absence of anti-SARS-CoV-2 ABT, indicating positive selection by endogenous immune responses, which were detected in several studies [8,61,71,92–94]. In other reports, testing for anti-SARS-CoV-2 antibodies revealed no evidence of an endogenous humoral immune response correlating with the appearance of mutations in the NTD supersite [75,95,96]. Notably, the lack of serological testing in certain investigations precludes any definitive conclusion regarding the potential involvement of an endogenous immune response in causing deletions in the NTD supersite [54,58,60,63,68,69,97]. The emergence of RDR2/RDR4 mutations either in combination with single RBM substitutions at positions E484 or Q493/S494 [8,9,13,54,60–62,66,68–71,75,93] or double substitutions at positions E484 and Q493/S494 [58,63,64], similar to the case reported here has also been observed during prolonged infection.

Given the considerable number of epitopes affected, it seems reasonable to posit that S:Delta141-4 E484K S494P may exhibit strong immune evasion properties. Accordingly, our findings demonstrate that mutant S:Delta141-4 E484K S494P highly significantly escapes neutralisation by sera of healthy controls obtained four weeks after their first vaccination. A comparable immune escape was observed with S:Omicron BA.1, yet not with S:S494P and S:E484K S494P. It is noteworthy that following two vaccinations, immune evasion was no longer detected with S:Delta141-4 E484K S494P, although it persisted with S:Omicron BA.1. This suggests that the combination of escape mutations in the NTD and RBM as observed in our patient may exert the most substantial impact during the initial phase of an anti-S immune response.

In the case reported here, the emergence of escape mutations in the NTD at a late stage of infection is unambiguous evidence of selective pressure by an endogenous antibody response. This is corroborated by a simultaneous increase in neutralising antibodies against S variants carrying bamlanivimab escape mutations, i.e., S:S494P, S:E484K S494P and S:Delta141-4 E484K S494P, in our patient. Apart from slightly elevated background values observed immediately after the administration of bamlanivimab, the utilisation of S antigens containing the bamlanivimab escape mutation S:S494P permitted the targeted detection of neutralising antibodies produced by the patient despite high bamlanivimab levels in serum samples. In contrast, when wt S was employed as a diagnostic antigen, the administration of bamlanivimab impeded the detection of an endogenous neutralising antibody response. Modest increases in neutralising antibody levels at the highest serum dilution tested may be indicative of an endogenous antibody response between days 40 and 60.

Of note, the patient´s polyclonal immune response between days 49 and 90 appeared to consist solely of IgM. This contrasts with the situation in immunocompetent individuals [98–100] and may reflect the gradual recovery of the humoral immune response from the suppressive effect of rituximab administered more than two months before SARS-CoV-2 infection.

The reaction to the patient’s first vaccination also indicated a long-lasting disturbance of the immune response. In contrast to healthy individuals with anamnestic infection [101,102], no prominent enhancement of IgG was observed; instead, IgM and IgA responses became evident. Low avidity and impaired affinity maturation of anti-S antibodies have been suggested to facilitate immune escape in prolonged infection [93]. The presented patient’s isolated IgM response and the lack of a detectable class switch to IgA or IgG during the acute infection stage may thus have initially facilitated the emergence of escape mutations prior to virus clearance, as observed by Truong and colleagues (2021) in a B-ALL patient [71]. On the other hand, virus clearance provides strong evidence of the neutralising capacity of the patient’s immune response. A highly active, neutralising IgM response was described by Jaki and colleagues (2023) as the most likely cause of viral clearance in a patient with prolonged infection and the emergence of resistance to the combined therapy with casirivimab and imdevimab [92]. Moreover, in some patients with advanced HIV, clearance of SARS-CoV-2 appears to be associated with strong increases in anti-spike IgM [103].

IgM antibodies derived from memory B cells potently neutralise SARS-CoV-2. On a molecular level, the activity of IgM is higher as compared to IgG and neutralising efficiency against variants is retained. These IgM-specific benefits are lost by conversion to IgG [104,105]. Vice versa, engineering high-affinity RBD-specific IgG1 antibodies to IgM results in a substantial increase in neutralising potency, reaching several hundred-fold, and a reduction in variant resistance [106]. Anti-S IgM was described as an active component in CCP [107,108]. Upon intranasal delivery, IgM efficiently protects mice from SARS-CoV-2 [106].

The important role of intra-spike cross-linking in the neutralising activity of IgG against SARS-CoV-2 and other viruses has been previously described [109–111]. In a humoral immune response dominated by pentameric IgM as observed in the case reported here, intra- and inter-spike cross-linking may become paramount for efficient neutralisation and retaining activity against escape variants to an even greater extent.

Interestingly, we observed only a marginal increase in inhibitory antibodies against S:S494P or S:E484K S494P in sVNT, which remained below 30% activity, albeit clearly positive results were obtained in the RBD IgM EIA using identical antigens. Thus, the intrinsic low-avidity binding of IgM to monomeric antigens, in comparison to multimeric antigenic structures present in the native S protein, may have prevented the detection of inhibitory IgM antibodies in the variant-specific sVNT. In addition, sVNTs may generally exhibit a lower correlation with a full virus neutralisation assay in convalescents as compared to vaccinees [47]. Poor reactivity with monomeric antigens may also explain the negative results obtained in the NTD EIA during the first episode of infection.

Concerning the period between the onset of antibody-mediated selective pressure and the first detection of antibody escape variants, it is remarkable that it took 15 days for the S:S494P bamlanivimab-resistant variant to emerge in our patient. In contrast, escape mutants carrying additional deletions and substitutions in the NTD and the RBD appeared to emerge concurrently with the onset of an endogenous, neutralising antibody response. This suggests that in the late phase of infection, the S:S494P variant had gained an enhanced capacity to respond rapidly to selective pressure owing to the genomic diversity accumulated throughout prolonged infection [7]. Moreover, dynamic differences in the observed spectrum of mutations may indicate the presence of a polymorphic virus population occupying discrete niches as described by Harari and colleagues (2022) [13]. This compartmentalisation may have contributed to the unsatisfactory therapeutic effect of the second remdesivir treatment our patient received [10]. In addition, in individuals with prolonged infection, nucleoside analogues like remdesivir may serve as potential co-factors in the emergence of escape mutants by increasing the pool of selectable S variants [97].

Compared to the first infection, the antibody response to the patient’s Omicron re-infection differed in several relevant aspects. First, SARS-CoV-2 neutralising antibodies elicited by the first infection and/or vaccination were present prior to re-infection; however, they failed to neutralise S:Delta141-4 E484K S494P and S Omicron BA.1. Accordingly, inhibitory antibodies directed against wt RBD and RBD S494P but not against RBD E484K S494P or RBD Omicron BA.1 were detected prior to re-infection. This observation reflects the immune escape mediated by the combination of substitutions S:E484K S494P in the pre-VOC context as discussed above and particularly the effect of the numerous escape mutations in S of the Omicron sublineages [33,78,112]. Reduced affinity maturation of the SARS-CoV-2-specific antibody response following the first infection and subsequent vaccination [113] may have contributed by mediating an IgG response with a reduced breadth and potency in our patient.

Second, re-infection with Omicron BA.1.18 led to a pronounced RBD-specific IgA response, while the IgM response was markedly diminished. Introducing the substitution S:E340K into the RBD, which interferes with the binding of sotrovimab [49,114], revealed that Omicron re-infection also elicited an increase in inhibitory antibodies and RBD-specific IgG. In addition, NTD-specific IgA and IgG responses were detected. Thus, the humoral immune response elicited by re-infection with Omicron more closely reflected the situation in breakthrough infections of vaccinated, immunocompetent individuals, which may explain the mild, self-limiting course of the disease [3,4,34].

There are several limitations to this study. The data presented here pertain to a single patient and can therefore only be applied to the entire group of immunocompromised patients to a limited extent. Previous diseases and specific forms of therapy used in this case may have strongly affected the course of infection and the humoral immune response against SARS-CoV-2. Moreover, it can be assumed that the initial immune response in B-cell-depleted patients may significantly differ in SARS-CoV-2 naive patients and individuals with pre-existing exposure due to vaccination or infection. Finally, the role of cellular and mucosal immunity was not analysed and requires further clarification, especially concerning viral clearance.

In conclusion, our data highlight the importance of a quantitatively and qualitatively insufficient endogenous humoral immune response as a driver of viral intra-host evolution. In particular, the data presented here emphasise that the onset of an endogenous anti-S IgM response in an immunocompromised patient with prolonged infection can create a critical situation favouring the sudden emergence and positive selection of viral antibody escape mutants after weeks of apparent genetic stability. During this unexpectedly dynamic phase of intra-host evolution, various variants with potential epidemiological relevance may emerge within a few days until virus elimination. To gain a deeper insight into this Janus-faced role of antibodies in the high-risk, immunocompromised host, it will be crucial to implement monitoring strategies based on whole genome sequencing and serological assays that can fully assess the patient- and variant-specific immune response in sufficient detail and temporal resolution.

## Supporting information

S1 Fig Detection of neutralising antibodies by in-house pVNT. Reactivity of the patient’s sera with wt S (a), S:S494P (b), S:E484K S494P (c), S:Delta141-4 E484K S494P (d) and S Omicron BA.1 (e) was determined by in-house pVNT. The results are presented as the mean +/- SD of four technical replicates and are given as a reduction in the GFP signal (%) as compared to the untreated control. The sera were tested at a dilution of 1:20, 1:80 and 1:320, as indicated. The values represent mean values of four individual experiments plus/minus standard deviation. The pVNT cut-off was set at ≥50% inhibition at a serum dilution of 1:20 (dotted horizontal line). Any values below zero were set to zero. The sample numbers refer to the date of sampling after the initial diagnosis (day 1), PC: positive control, pooled sera of individuals four weeks after the second vaccination, NC: negative control, pooled sera of individuals before the first vaccination.
(TIFF)

S2 Fig Neutralisation of S variants by sera obtained from healthy vaccinees. Neutralising antibodies were determined by pVNT in serum samples obtained from nine vaccinees at four time points: immediately before the first vaccination (t0), four weeks after the first vaccination (t1), four weeks after the second vaccination (t2) and three months after the second vaccination (t3). The sera were tested at a dilution of 1:20 in the pVNT with the antigens indicated. The values represent mean values of the tested vaccinees (four individual experiments each) plus/minus standard deviation. The cut-off was set at 50% inhibition. Values below zero were set to zero. The number of sera that were positive in the pVNT is shown above the bars. PC: positive control (serum pool obtained after second vaccination), NC: negative control (serum pool obtained before vaccination). Values below zero were set to zero.
(TIFF)

S3 Fig Detection of RBD-reactive inhibitory antibodies in variant-specific sVNT. Sera were tested at a dilution of 1:20 in the in-house sVNT with the antigens indicated on the x-axis. Results are given as the mean of two independent experiments, the inhibitory activity is depicted by a heat map and given in absolute numbers (% inhibition). Blanks are crossed out. The cut-off was set at 25% inhibition. PC: positive control (serum pool obtained after second vaccination), NC: negative control (serum pool obtained before vaccination). Values below zero were set to zero.
(TIFF)

S4 Fig Serum reactivity in variant-specific RBD- and NTD-EIA. Sera were tested at a dilution of 1:100 in the Ig class capture EIA with the antigens indicated on the x-axis. The detection of RBD-reactive IgG, IgM and IgA is depicted in panels a, b, c; the detection of NTD-reactive IgG, IgM and IgA is shown in panels e, f, g. Results are given as the mean of two independent experiments, the signal strength is depicted by a heat map and given in absolute numbers (rlu). Blanks are crossed out. The cut-off was set at 300 rlu in the RBD-EIA and at 100 rlu in the NTD-EIA. PC: positive control (serum pool obtained after second vaccination), NC: negative control (serum pool obtained before vaccination). Values below zero were set to zero.
(TIFF)

S1 Table a) List of expression plasmids. b) List of primers.
(DOCX)

S2 Table a) Development of the CD19+ B cell population over the observation period. b) Development of the CD3+ T cell population over the observation period. c) Development of the CD4+ T cell population over the observation period. d) Development of the CD8+ T cell population over the observation period. e) Immunoglobulin levels over the observation period.
(DOCX)

S3 Table List of specimens, accession numbers, PANGOLIN lineages and mutations in the spike consensus sequence. (DOCX)

S4 Table Frequency of mutations on read level. All raw sequence data are deposited at the NCBI SRA under bioproject number PRJNA1120916.
(XLSX)

S5 Table Source data for figures. (XLSX)
