# Supplementary figures and images for: Characterisation of the antibody-mediated selective pressure driving intra-host evolution of SARS-CoV-2 in prolonged infection
Source: PLoS Pathog. 2024 Oct 15;20(10):e1012624. doi: 10.1371/journal.ppat.1012624 (PMC11508484; doi:10.1371/journal.ppat.1012624)

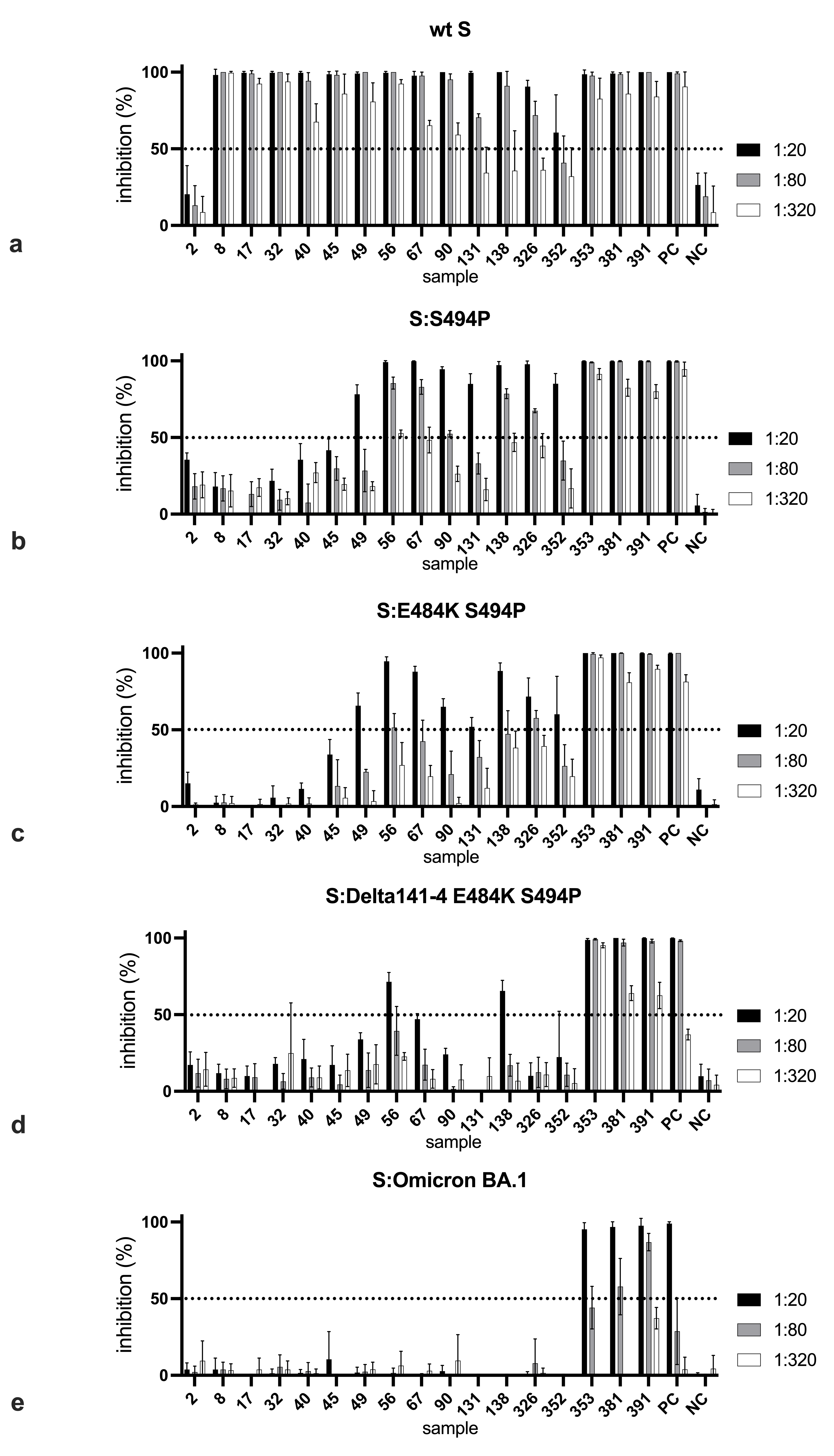

Supplement: S1 Fig — Reactivity of the patient’s sera with wt S (a), S:S494P (b), S:E484K S494P (c), S:Delta141-4 E484K S494P (d) and S Omicron BA.1 (e) was determined by in-house pVNT. The results are presented as the mean +/- SD of four technical replicates and are given as a reduction in the GFP signal (%) as compared to the untreated control. The sera were tested at a dilution of 1:20, 1:80 and 1:320, as indicated. The values represent mean values of four individual experiments plus/minus standard deviation. The pVNT cut-off was set at ≥50% inhibition at a serum dilution of 1:20 (dotted horizontal line). Any values below zero were set to zero. The sample numbers refer to the date of sampling after the initial diagnosis (day 1), PC: positive control, pooled sera of individuals four weeks after the second vaccination, NC: negative control, pooled sera of individuals before the first vaccination. (TIFF) [file ppat.1012624.s001.tiff]

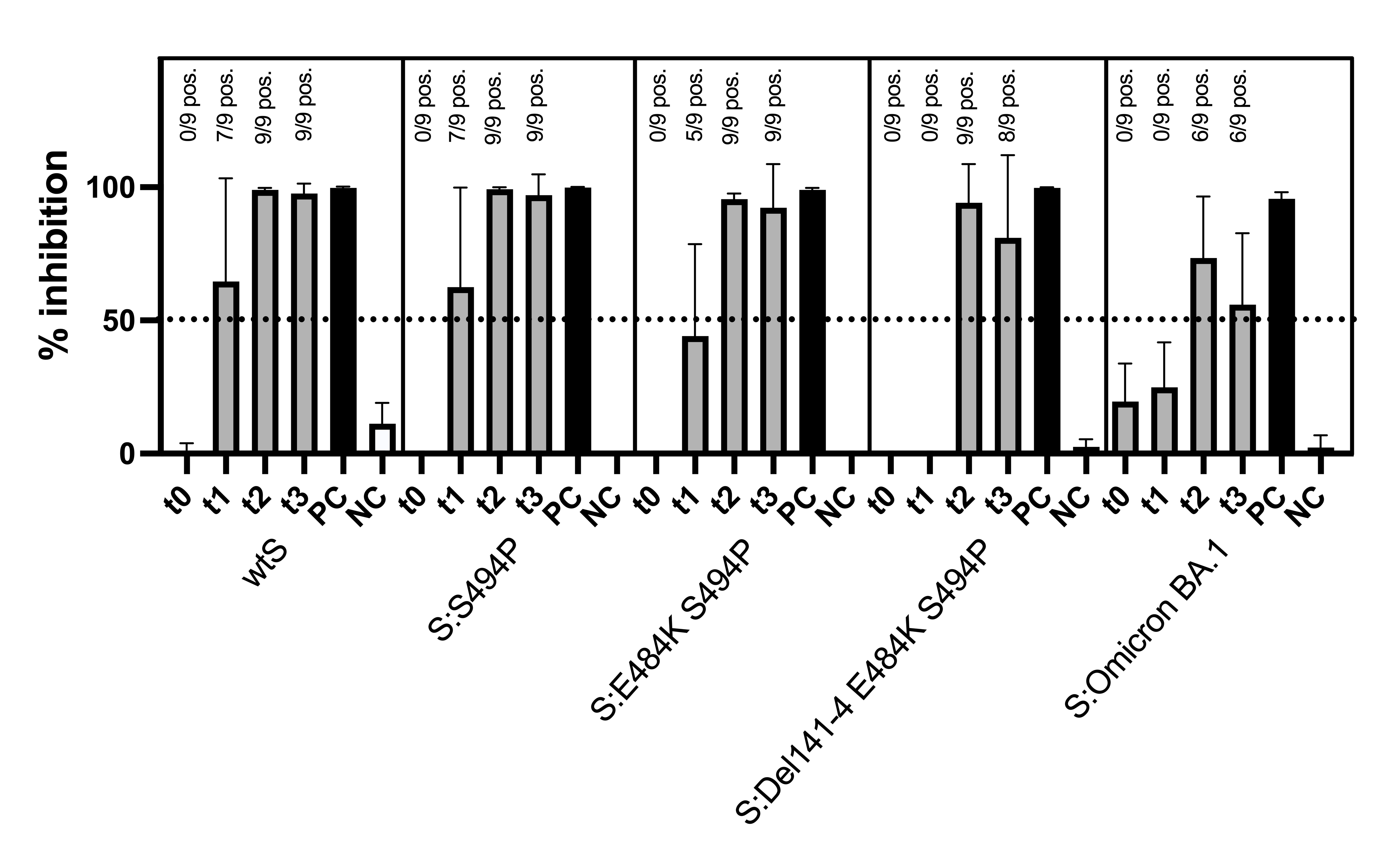

Supplement: S2 Fig — Neutralising antibodies were determined by pVNT in serum samples obtained from nine vaccinees at four time points: immediately before the first vaccination (t0), four weeks after the first vaccination (t1), four weeks after the second vaccination (t2) and three months after the second vaccination (t3). The sera were tested at a dilution of 1:20 in the pVNT with the antigens indicated. The values represent mean values of the tested vaccinees (four individual experiments each) plus/minus standard deviation. The cut-off was set at 50% inhibition. Values below zero were set to zero. The number of sera that were positive in the pVNT is shown above the bars. PC: positive control (serum pool obtained after second vaccination), NC: negative control (serum pool obtained before vaccination). Values below zero were set to zero. (TIFF) [file ppat.1012624.s002.tiff]

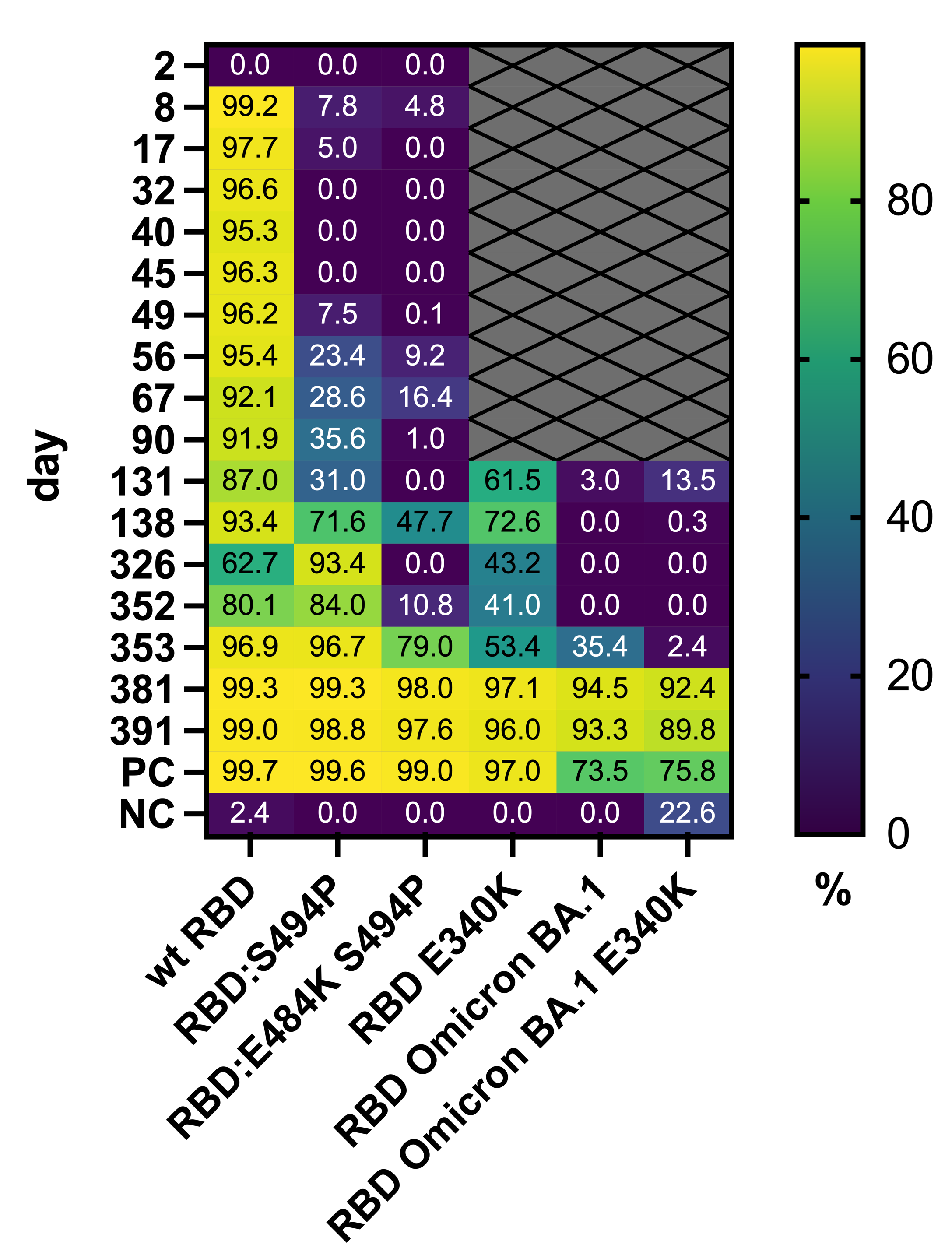

Supplement: S3 Fig — Sera were tested at a dilution of 1:20 in the in-house sVNT with the antigens indicated on the x-axis. Results are given as the mean of two independent experiments, the inhibitory activity is depicted by a heat map and given in absolute numbers (% inhibition). Blanks are crossed out. The cut-off was set at 25% inhibition. PC: positive control (serum pool obtained after second vaccination), NC: negative control (serum pool obtained before vaccination). Values below zero were set to zero. (TIFF) [file ppat.1012624.s003.tiff]

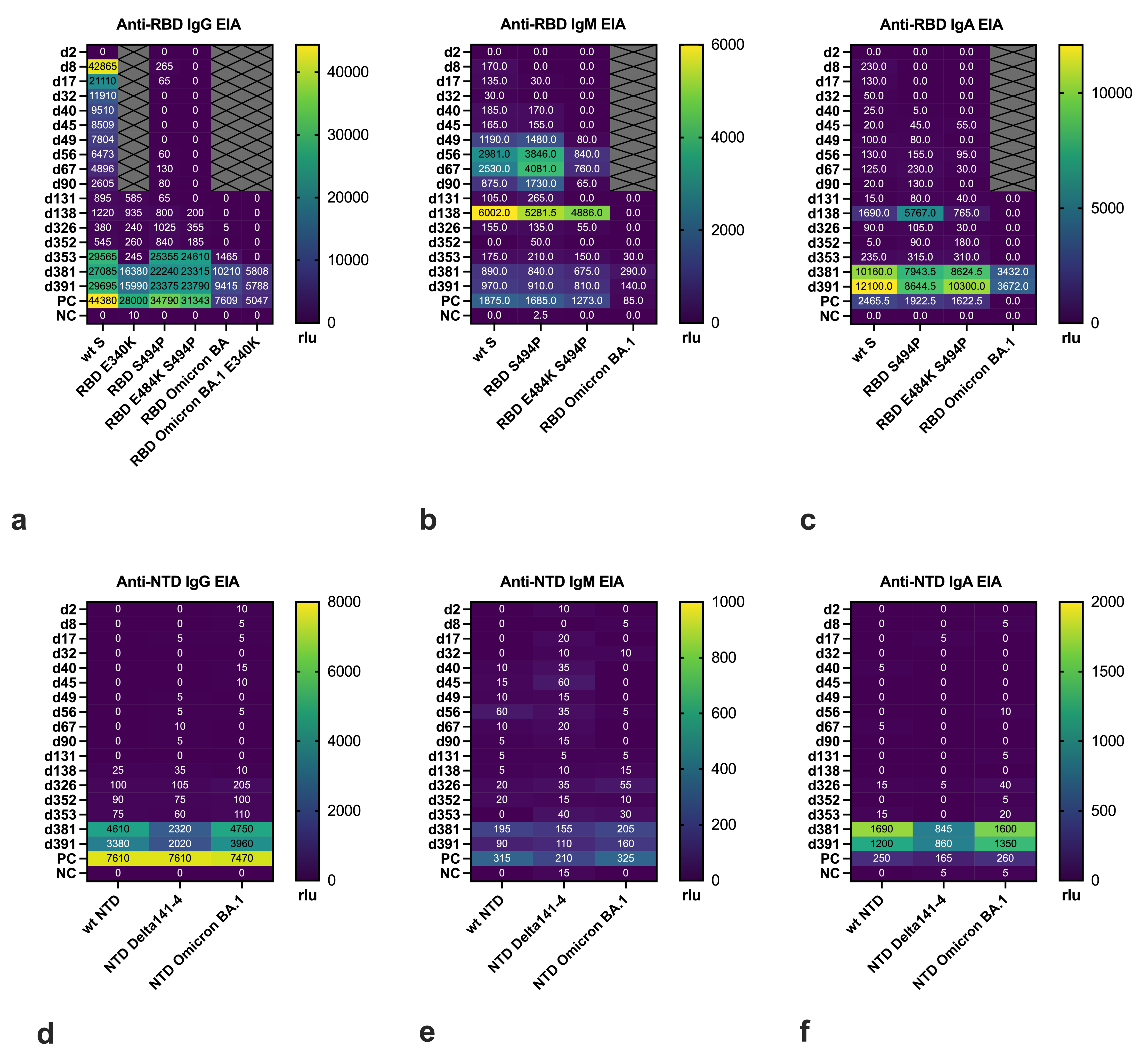

Supplement: S4 Fig — Sera were tested at a dilution of 1:100 in the Ig class capture EIA with the antigens indicated on the x-axis. The detection of RBD-reactive IgG, IgM and IgA is depicted in panels a, b, c; the detection of NTD-reactive IgG, IgM and IgA is shown in panels e, f, g. Results are given as the mean of two independent experiments, the signal strength is depicted by a heat map and given in absolute numbers (rlu). Blanks are crossed out. The cut-off was set at 300 rlu in the RBD-EIA and at 100 rlu in the NTD-EIA. PC: positive control (serum pool obtained after second vaccination), NC: negative control (serum pool obtained before vaccination). Values below zero were set to zero. (TIFF) [file ppat.1012624.s004.tiff]
