# Supplementary material for: Characterisation of the antibody-mediated selective pressure driving intra-host evolution of SARS-CoV-2 in prolonged infection
Source: PLoS Pathog. 2024 Oct 15;20(10):e1012624. doi: 10.1371/journal.ppat.1012624 (PMC11508484; doi:10.1371/journal.ppat.1012624)
Supplement: S1 Table — a) List of expression plasmids. b) List of primers. (DOCX) [file ppat.1012624.s005.docx]

**S1 Table**

**a) List of expression plasmids.**

| **Vector** | **Insert** |
| --- | --- |
| pCG1-SARS-2-S-Delta1253 | wt S protein with C-terminal 21aa deletion |
| pCG1-SARS-2-S-494P-Delta1253 | wt S protein with C-terminal 21aa deletion and substitution S494P |
| pCG1-SARS-2-S-484K-494P-Delta1253 | wt S protein with C-terminal 21aa deletion and substitutions E484K and S494P |
| pCG1-SARS-2-S-Delta141-4-484K-494P-Delta1253 | wt S protein with C-terminal 21aa deletion, deletion 141-4, and substitutions E484K and S494P |
| pcDNA3.1 SARS-2 Omicron | Omicron BA.1 S protein with C-terminal 21aa deletion |
| pEN-secNL-RBD | secreted RBD fragment of S protein, aa 319 to aa 541, wt sequence, N-terminal NLuc tag |
| pEN-secNL-RBD-E340K | secreted RBD fragment of S protein, aa 319 to aa 541, wt sequence with substitution E340K, N-terminal NLuc tag |
| pEN-secNL-RBD-494P | secreted RBD fragment of S protein, aa 319 to aa 541, wt sequence with substitution S494P, N-terminal NLuc tag |
| pEN-secNL-RBD-484K-494P | secreted RBD fragment of S protein, aa 319 to aa 541, wt sequence with substitutions E484K and S494P, N-terminal NLuc tag |
| pEN-secNL-RBD-Omicron | secreted RBD fragment of S protein, aa 319 to aa 541, Omicron BA.1 sequence, N-terminal NLuc tag |
| pEN-secNL-RBD-Omicron-E340K | secreted RBD fragment of S protein, aa 319 to aa 541, Omicron BA.1 sequence with additional substitution E340K, N-terminal NLuc tag |
| pEN-secNL-NTD | secreted NTD fragment of S protein, aa 15 to aa 307 |
| pEN-secNL-NTD-Delta141-4 | secreted NTD fragment of S protein, aa 15 to aa 307 with deletion 141-4, N-terminal NLuc tag |
| pEN-secNL-NTD-Omicron | secreted NTD fragment of S Omicron BA.1 protein, aa 15 to aa 307 |

**b) List of primers.**

| **Primer** | **Sequence 5´-3´** |
| --- | --- |
| CG1-S-Bam fwd | GGG CGA ATT CGG ATC CGC CAC C*AT G* |
| CG1-S-Age bwd | ATGCCGTTGAACCGGTAGGCCATCTGC |
| CG1-494P fwd | CTG CAG CCC TAC GGC TTT CAG CCC AC |
| CG1-494P bwd | GCC GTA GGG CTG CAG TGG GAA GTA GCA |
| CG1-484K fwd | C GGC GTG AAA GGC TTC AAC TGC TAC TTC CCA CT |
| CG1-484K bwd | AA GCC TTT CAC GCC GTT ACA AGG GGT GCT GCC G |
| CG1-Delta141-4 fwd | GAC CCC TTC TAT CAC AAG AAC AAC AAG AGC TGG ATG GAA AGC GAG |
| CG1-Delta141-4 bwd | GTG ATA GAA GGG GTC GTT GCA GAA CTG GAA CTC G |
| RBD-BamHI_2 fwd | C ATT CTG GCG GGA TCC CGG GTG CAG CCC ACC GAA TCC ATC |
| RBD-NotI bwd | TC TAG AGT CGC GGC CGC TTA TCA GAA GTT CAC GCA TTT GTT CTT CAC GAG |
| secNL fwd | CGT CAG ATC CGC TAG C ATG AAC TCC TTC TCC ACA AGC |
| wt E340K fwd | TTC GGC AAG GTG TTC AAT GCC ACC AGA TTC GCC |
| wt E340K bwd | GAA CAC CTT GCC GAA GGG GCA CAG ATT GGT |
| Omicron E340K fwd | TTC GAT AAG GTG TTC AAT GCC ACC AGA TTC GCC |
| Omicron E340K bwd | GAA CAC CTT ATC GAA GGG GCA CAG ATT GGT |
| S15-BamH1 fwd | C ATT CTG GCG GGA TCC TGT GTG AAC CTG ACC ACA AGA ACC |
| S307-Not1 bwd | TC TAG AGT CGC GGC CGC TTA TCA GGT GAA GGA CTT CAG GGT GCA C |
