# Supplementary material for: Characterisation of the antibody-mediated selective pressure driving intra-host evolution of SARS-CoV-2 in prolonged infection
Source: PLoS Pathog. 2024 Oct 15;20(10):e1012624. doi: 10.1371/journal.ppat.1012624 (PMC11508484; doi:10.1371/journal.ppat.1012624)
Supplement: S2 Table — a) Development of the CD19+ B cell population over the observation period. b) Development of the CD3+ T cell population over the observation period. c) Development of the CD4+ T cell population over the observation period. d) Development of the CD8+ T cell population over the observation period. e) Immunoglobulin levels over the observation period. (DOCX) [file ppat.1012624.s006.docx]

**S2 Table**

**a) Development of the CD19+ B cell population over the observation period.**

| **Day** | **% (Normal Value 60 – 85%)** | **Cells/µL (Normal Value 100 – 500)** |
| --- | --- | --- |
| -37 | 0.01 | 0.03 |
| 98 | 0.36 | 0.12 |
| 194 | 2.26 | 8.19 |
| 221 | 3.94 | 23.65 |
| 264 | 8.08 | 45.56 |
| 297 | 13.43 | 73.96 |
| 326 | 16.40 | 96.45 |
| 391 | 18.96 | 102.08 |
| 438 | 23.43 | 148.63 |
| 466 | 27.71 | 121.62 |

D1 designates the day of the first SARS-CoV-2 infection diagnosis.

**b) Development of the CD3+ T cell population over the observation period.**

| **Day** | **% (Normal Value 60 – 85%)** | **Cells/µl (Normal Value 700 – 2100)** |
| --- | --- | --- |
| -37 | 86.57 | 336 |
| 98 | 76.16 | 240 |
| 194 | 70.66 | 256 |
| 221 | 63.10 | 378 |
| 264 | 68.44 | 385 |
| 297 | 63.91 | 351 |
| 326 | 66.04 | 388 |
| 391 | 61.16 | 329 |
| 438 | 58.56 | 371 |
| 466 | 57.00 | 250 |

D1 designates the day of the first SARS-CoV-2 infection diagnosis.

**c) Development of the CD4+ T cell population over the observation period.**

| **Day** | **% (Normal Value 29 – 59%)** | **Cells/µl (Normal Value 450 – 2000)** |
| --- | --- | --- |
| -37 | 19.5 | 36.9 |
| 98 | 20.2 | 60.5 |
| 194 | 14.7 | 53.6 |
| 221 | 15.5 | 93.2 |
| 264 | 13.5 | 75.8 |
| 297 | 15.7 | 86.6 |
| 326 | 14.5 | 84.9 |
| 391 | 11.5 | 61.9 |
| 438 | 11.9 | 75.8 |
| 466 | 14.8 | 62.1 |

D1 designates the day of the first SARS-CoV-2 infection diagnosis.

**S2 Table d) Development of the CD8+ T cell population over the observation period.**

| **Day** | **% (Normal Value 19 – 48%)** | **Cells/µl (Normal Value 250 – 1700)** |
| --- | --- | --- |
| -37 | 76.9 | 298 |
| 98 | 55.8 | 180 |
| 194 | 54.2 | 196 |
| 221 | 45.9 | 275 |
| 264 | 52.8 | 298 |
| 297 | 47.1 | 259 |
| 326 | 49.5 | 291 |
| 391 | 47.9 | 257 |
| 438 | 44.5 | 282 |
| 466 | 41.8 | 183 |

D1 designates the day of the first SARS-CoV-2 infection diagnosis.

**S2 Table e) Immunoglobulin levels over the observation period.**

| **Day** | **IgG mg/dL (Normal Value 700 – 1600)** | **IgM mg/dL (Normal Value 40 – 230)** | **IgA mg/dL (Normal Value 70 – 400)** |
| --- | --- | --- | --- |
| -21 | 458 | 17 | 84 |
| 4 | 491 | 22 | n.a. |
| 66 | 808 | 26 | 48 |
| 199 | 961 | 43 | 32 |
| 351 | 1725 | 76 | 162 |
| 391 | 1812 | 62 | 200 |
| 486 | 1782 | 57 | 226 |

D1 designates the day of the first SARS-CoV-2 infection diagnosis.
