# Supplementary material for: Characterisation of the antibody-mediated selective pressure driving intra-host evolution of SARS-CoV-2 in prolonged infection
Source: PLoS Pathog. 2024 Oct 15;20(10):e1012624. doi: 10.1371/journal.ppat.1012624 (PMC11508484; doi:10.1371/journal.ppat.1012624)
Supplement: S3 Table — (DOCX) [file ppat.1012624.s007.docx]

**S3 Table.** **List of specimens, GISAID accession numbers, PANGOLIN lineages and mutations in the spike sequence of virus isolated from the patient.**

| **First episode of SARS-CoV-2 infection** | | | |
| --- | --- | --- | --- |
| **Specimen** | **GISAID**  **accession no.** | **PANGOLIN**  **lineage** | **Mutations in spike** |
| *d1** | EPI_ISL_1726855 | B.1.177.81 | A222V, D614G |
| *d8* | EPI_ISL_17817499 | B.1.177.81 | A222V, D614G |
| *d12* | EPI_ISL_17817834 | B.1.177.81 | A222V, D614G |
| *d15* | EPI_ISL_17817920 | B.1.177.81 | A222V, S494P, D614G |
| *d16* | EPI_ISL_17817921 | B.1.177.81 | A222V, S494P, D614G |
| *d20* | EPI_ISL_1353439 | B.1.177.81 | A222V, S494P, D614G |
| *d26* | EPI_ISL_17817922 | B.1.177.81 | A222V, S494P, D614G |
| *d27* | EPI_ISL_17817927 | B.1.177.81 | A222V, S494P, D614G |
| *d30* | EPI_ISL_17817937 | B.1.177.81 | A222V, S494P, D614G |
| *d36* | EPI_ISL_1435916 | B.1.177.81 | A222V, S494P, D614G |
| *d40* | EPI_ISL_17817938 | B.1.177.81 | A222V, S494P, D614G |
| *d42* | EPI_ISL_17817939 | B.1.177.81 | A222V, S494P, D614G |
| *d45, sample1* | EPI_ISL_1567101 | B.1.177.81 | L141del, G142del, V143del, Y144F, A222V, S494P, D614G |
| *d45, sample2* | EPI_ISL_17817940 | B.1.177.81 | T19I, L141del, G142del, V143del, Y144F, A222V, S494P, D614G |
| *d47, sample1* | EPI_ISL_17817941 | B.1.177.81 | F140del, A222V, E484K, S494P, D614G |
| *d47, sample2* | EPI_ISL_17817942 | B.1.177.81 | F140del, A222V, E484K, S494P, D614G |
| *d48, sample1* | EPI_ISL_1643829 | B.1.177.81 | L141del, G142del,V143del, Y144del, A222V, L242del, A243del, L244del, E484K, S494P, D614G |
| *d48, sample2* | EPI_ISL_17817943 | B.1.177.81 | L141del, G142del, V143del, Y144del, A222V, E484K, S494P, D614G |
| *d52, sample1* | EPI_ISL_17817944 | B.1.177.81 | L141del, G142del, V143del, Y144del, A222V, E484K, S494P, D614G |
| *d52, sample2* | EPI_ISL_17817945 | B.1.177.81 | L141del, G142del, V143del, Y144del, A222V, E484K, S494P, D614G |
| *d54* | EPI_ISL_17817946 | B.1.177.81 | L141del, G142del, V143del, Y144del, A222V, E484K, S494P, D614G |
| **Second episode of SARS-CoV-2 infection** | | | |
| d352 | EPI_ISL_9740094 | BA.1.18 | A67V, H69del, V70del, T95I, G142D, V143del, Y144del, Y145del, N211del, L212I, ins214EPE, G339D, S371L, S373P, S375F, K417N, N440K, G446S, S477N, T478K, E484A, Q493R, G496S, Q498R, N501Y, Y505H, T547K, D614G, H655Y, N679K, P681H, N764K, D796Y, N856K, Q954H, N969K, L981F |
